# Supplementary material for: Effects of mechanical interventions in the management of knee osteoarthritis: protocol for an OA Trial Bank systematic review and individual participant data meta-analysis
Source: BMJ Open. 2021 Feb 5;11(2):e043026. doi: 10.1136/bmjopen-2020-043026 (PMC7925922; doi:10.1136/bmjopen-2020-043026)
Supplement: Supplementary data [file bmjopen-2020-043026supp001.pdf]

Appendix 1. Search Terms

Medline (OVID)

(Osteoarthritis, Knee/ OR ((Knee Joint/ OR Knee/ OR Patellofemoral Joint/ OR knee function/) AND Osteoarthritis/) OR (((knee* OR femorotib* OR tibiofemor* OR patellofemoral* OR femoropatell* OR gon) ADJ6 (osteoarth* OR arthrit* OR arthrosis OR oa)) OR (femor* ADJ3 (tibi* OR patell*) ADJ6 (osteoarth* OR arthrit* OR arthrosis OR oa)) OR gonarthr*).ab,ti.) AND (Orthotic Devices/ OR Braces/ OR Foot Orthoses/ OR Shoes/ OR Canes/ OR Crutches/ OR Athletic Tape/ OR (orthos* OR orthotic* OR brace* OR bracing* OR ((orthop* OR foot-worn) ADJ3 (support* OR device* OR applian* OR apparat* OR platform*)) OR shoe* OR crutch* OR cane OR canes OR tape OR taping OR kinesiotap* OR KneeBrac* OR ((mechanical* OR biomechan*) ADJ3 (intervent* OR treat* OR therap*)) OR insole* OR sleeve* OR footwear* OR foot-wear* OR walking-stick* OR walking-aid* OR ((elastic* OR adhesi*) ADJ3 bandage*)).ab,ti.) AND (Exp Controlled clinical trial/ OR "Double-Blind Method"/ OR "Single-Blind Method"/ OR "Random Allocation"/ OR (random* OR factorial* OR crossover* OR cross over* OR placebo* OR ((doubl* OR singl*) ADJ blind*) OR assign* OR allocat* OR volunteer* OR trial OR groups).ab,ti.) NOT (exp Animals/ NOT Humans/)

embase.com

('knee osteoarthritis'/de OR 'patellofemoral arthritis'/de OR (('knee pain'/de OR 'knee'/de OR 'patellofemoral joint'/de OR 'knee function'/de) AND osteoarthritis/de) OR 'Knee Injury and Osteoarthritis Outcome'/de OR 'knee arthritis'/de OR (((knee* OR femorotib* OR tibiofemor* OR patellofemoral* OR femoropatell* OR gon) NEAR/6 (osteoarth* OR arthrit* OR arthrosis OR oa)) OR (femor* NEAR/3 (tibi* OR patell*) NEAR/6 (osteoarth* OR arthrit* OR arthrosis OR oa)) OR gonarthr*):ab,ti) AND ('orthosis'/de OR orthotics/de OR brace/de OR 'knee orthosis'/exp OR 'inshoe orthosis'/de OR 'leg orthosis'/de OR 'foot orthosis'/exp OR 'walking orthosis'/de OR 'shoe'/de OR 'cane'/de OR crutch/exp OR 'walking aid'/exp OR 'orthopedic shoe'/exp OR 'athletic tape'/de OR 'kinesiotape'/de OR 'kinesio tape'/de OR 'kinesio taping'/de OR 'kinesiotaping'/de OR 'bandaging technique'/de OR 'elastic adhesive bandage'/de OR (orthos* OR orthotic* OR brace* OR bracing* OR ((orthop* OR foot-worn) NEAR/3 (support* OR device* OR applian* OR apparat* OR platform*)) OR shoe* OR crutch* OR cane OR canes OR tape OR taping OR kinesiotap* OR KneeBrac* OR ((mechanical* OR biomechan*) NEAR/3 (intervent* OR treat* OR therap*)) OR insole* OR sleeve* OR footwear* OR foot-wear* OR walking-stick* OR walking-aid* OR ((elastic* OR adhesi*) NEAR/3 bandage*)):ab,ti) AND ('Controlled clinical trial'/exp OR 'Crossover procedure'/de OR 'Double-blind procedure'/de OR 'Single-blind procedure'/de OR (random* OR factorial* OR crossover* OR (cross NEXT/1 over*) OR placebo* OR ((doubl* OR singl*) NEXT/1 blind*) OR assign* OR allocat* OR volunteer* OR trial OR groups):ab,ti) NOT ([animals]/lim NOT [humans]/lim)

CINAHL EBSCOhost

(Osteoarthritis, Knee+ OR ((Knee Joint+ OR Knee+ OR Patellofemoral Joint+ OR knee function+) AND Osteoarthritis+) OR (((knee* OR femorotib* OR tibiofemor* OR patellofemoral* OR femoropatell* OR gon) N5 (osteoarth* OR arthrit* OR arthrosis OR oa)) OR (femor* N2 (tibi* OR patell*) N5 (osteoarth* OR arthrit* OR arthrosis OR oa)) OR gonarthr*)) AND (Orthotic Devices+ OR Braces+ OR Foot Orthoses+ OR Shoes+ OR Canes+ OR Crutches+ OR Athletic Tape+ OR (orthos* OR orthotic* OR brace* OR bracing* OR ((orthop* OR foot-worn) N2 (support* OR device* OR applian* OR apparat* OR platform*)) OR shoe* OR crutch* OR cane OR canes OR tape OR taping OR kinesiotap* OR KneeBrac* OR ((mechanical* OR biomechan*) N2 (intervent* OR treat* OR therap*)) OR insole* OR sleeve* OR footwear* OR foot-wear* OR walking-stick* OR walking-aid* OR ((elastic* OR adhesi*) N2 bandage*))) AND (MH Controlled clinical trial+ OR "Double-Blind Method+" OR "Single-Blind Method+" OR "Random Allocation+" OR (random* OR factorial* OR crossover* OR cross over* OR placebo* OR ((doubl* OR singl*) N1 blind*) OR assign* OR allocat* OR volunteer* OR trial OR groups)) NOT (MH Animals+ NOT Humans+)

Cochrane CENTRAL registry of trials

((((knee* OR femorotib* OR tibiofemor* OR patellofemoral* OR femoropatell* OR gon) NEAR/6 (osteoarth* OR arthrit* OR arthrosis OR oa)) OR (femor* NEAR/3 (tibi* OR patell*) NEAR/6 (osteoarth* OR arthrit* OR arthrosis OR oa)) OR gonarthr*):ab,ti) AND ((orthos* OR orthotic* OR brace* OR bracing* OR ((orthop* OR foot next worn) NEAR/3 (support* OR device* OR applian* OR apparat* OR platform*)) OR shoe* OR crutch* OR cane OR canes OR tape OR taping OR kinesiotap* OR KneeBrac* OR ((mechanical* OR biomechan*) NEAR/3 (intervent* OR treat* OR therap*)) OR insole* OR sleeve* OR footwear* OR foot next wear* OR walking next stick* OR walking next aid* OR ((elastic* OR adhesi*) NEAR/3 bandage*)):ab,ti)

Web of science Core Collection

TS=(((((knee* OR femorotib* OR tibiofemor* OR patellofemoral* OR femoropatell* OR gon) NEAR/5 (osteoarth* OR arthrit* OR arthrosis OR oa)) OR (femor* NEAR/2 (tibi* OR patell*) NEAR/5 (osteoarth* OR arthrit* OR arthrosis OR oa)) OR gonarthr*)) AND ((orthos* OR orthotic* OR brace* OR bracing* OR ((orthop* OR foot-worn) NEAR/2 (support* OR device* OR applian* OR apparat* OR platform*)) OR shoe* OR crutch* OR cane OR canes OR tape OR taping OR kinesiotap* OR KneeBrac* OR ((mechanical* OR biomechan*) NEAR/2 (intervent* OR treat* OR therap*)) OR insole* OR sleeve* OR footwear* OR foot-wear* OR walking-stick* OR walking-aid* OR ((elastic* OR adhesi*) NEAR/2 bandage*))) AND (random* OR factorial* OR crossover* OR cross over* OR placebo* OR ((doubl* OR singl*) N1 blind*) OR assign* OR allocat* OR volunteer* OR trial OR groups))
