# Supplementary material for: Effects of mechanical interventions in the management of knee osteoarthritis: protocol for an OA Trial Bank systematic review and individual participant data meta-analysis
Source: BMJ Open. 2021 Feb 5;11(2):e043026. doi: 10.1136/bmjopen-2020-043026 (PMC7925922; doi:10.1136/bmjopen-2020-043026)
Supplement: Author's manuscript [file bmjopen-2020-043026.draft_revisions.pdf]

BMJ Open is committed to open peer review. As part of this commitment we make the peer review history of every article we publish publicly available.

When an article is published we post the peer reviewers' comments and the authors' responses online. We also post the versions of the paper that were used during peer review. These are the versions that the peer review comments apply to.

The versions of the paper that follow are the versions that were submitted during the peer review process. They are not the versions of record or the final published versions. They should not be cited or distributed as the published version of this manuscript.

BMJ Open is an open access journal and the full, final, typeset and author-corrected version of record of the manuscript is available on our site with no access controls, subscription charges or pay-per-view fees (http://bmjopen.bmj.com).

If you have any questions on BMJ Open's open peer review process please email info.bmjopen@bmj.com

BMJ Open

The effects of mechanical interventions in the management of knee osteoarthritis: protocol for an OA Trial Bank systematic review and individual participant data meta-analysis

Journal:	*BMJ Open*
Manuscript ID	bmjopen-2020-043026
Article Type:	Protocol
Date Submitted by the Author:	22-Jul-2020
Complete List of Authors:	Macri, Erin; Erasmus MC, General Practice; The University of British Columbia, Family Practice<br>Callaghan, Michael; Manchester Metropolitan University, Health Professions<br>van Middelkoop, Marienke; Erasmus MC, General Practice<br>Hattle, Miriam; Keele University<br>Bierma-Zeinstra, Sita; Erasmus University Medical Centre, Department of General Practice
Keywords:	Knee < ORTHOPAEDIC & TRAUMA SURGERY, RHEUMATOLOGY, REHABILITATION MEDICINE

SCHOLARONE™
Manuscripts

I, the Submitting Author has the right to grant and does grant on behalf of all authors of the Work (as defined in the below author licence), an exclusive licence and/or a non-exclusive licence for contributions from authors who are: i) UK Crown employees; ii) where BMJ has agreed a CC-BY licence shall apply, and/or iii) in accordance with the terms applicable for US Federal Government officers or employees acting as part of their official duties; on a worldwide, perpetual, irrevocable, royalty-free basis to BMJ Publishing Group Ltd ("BMJ") its licensees and where the relevant Journal is co-owned by BMJ to the co-owners of the Journal, to publish the Work in this journal and any other BMJ products and to exploit all rights, as set out in our licence.

The Submitting Author accepts and understands that any supply made under these terms is made by BMJ to the Submitting Author unless you are acting as an employee on behalf of your employer or a postgraduate student of an affiliated institution which is paying any applicable article publishing charge ("APC") for Open Access articles. Where the Submitting Author wishes to make the Work available on an Open Access basis (and intends to pay the relevant APC), the terms of reuse of such Open Access shall be governed by a Creative Commons licence – details of these licences and which Creative Commons licence will apply to this Work are set out in our licence referred to above.

Other than as permitted in any relevant BMJ Author's Self Archiving Policies, I confirm this Work has not been accepted for publication elsewhere, is not being considered for publication elsewhere and does not duplicate material already published. I confirm all authors consent to publication of this Work and authorise the granting of this licence.

For peer review only

The effects of mechanical interventions in the management of knee osteoarthritis: protocol for an OA Trial Bank systematic review and individual participant data meta-analysis

Erin M Macri[1] e.macri@erasmusmc.nl
Michael J Callaghan[2] michael.callaghan@mmu.ac.uk
Marienke van Middelkoop[1] m.vanmiddelkoop@erasmusmc.nl
Miriam Hattle[3] m.s.hattle@keele.ac.uk
Sita MA Bierma-Zeinstra[1] s.bierma-zeinstra@erasmusmc.nl

Affiliations: 1. Erasmus MC, Rotterdam, NL; 2. Manchester Metropolitan University, Manchester, UK; 3. Keele University, Staffordshire, UK

Correspondance:
EM Macri
Erasmus University Medical Center Rotterdam
Na1907, Wytemaweg 80, 3015 CN Rotterdam, The Netherlands

For peer review only

For peer review only - http://bmjopen.bmj.com/site/about/guidelines.xhtml

Abstract

Introduction: Knee osteoarthritis (OA) is a prevalent and disabling musculoskeletal condition. Biomechanical factors may play a key role in the etiology of knee OA, therefore a broad class of interventions involves the application or wear of devices designed to mechanically support knees with OA. These include gait aids, bracing, taping, orthotics, and footwear. The literature regarding efficacy of mechanical interventions has been conflicting or inconclusive, and this may be because certain subgroups with knee OA respond better to mechanical interventions. Our primary aim is to identify subgroups with knee OA who respond favourably to mechanical interventions. *Methods and analysis:* We will conduct a systematic review to identify randomized clinical trials of any mechanical intervention for the treatment of knee OA. We will invite lead authors of eligible studies to share individual participant data (IPD). We will perform an IPD meta-analysis for each type of mechanical intervention to evaluate efficacy, with our main outcome being pain. We will then evaluate four potential treatment effect-modifiers using a two-stage approach. If data permit, we will also evaluate whether biomechanics mediate the effects of mechanical interventions on pain in knee OA.

Ethics and dissemination: No new data will be collected in this study. We will adhere to institutional, national and international regulations regarding the secure and confidential sharing of IPD, addressing ethics as indicated. We will disseminate findings via international conferences, open-source publication in peer-reviewed journals, and summaries posted on websites serving the public and clinicians.

Registration: PROSPERO CRD42020155466

For peer review only - http://bmjopen.bmj.com/site/about/guidelines.xhtml

Strengths and limitations of this study

- We designed our protocol in collaboration with the OA Trial Bank, an internationally-recognized organization with considerable IPD experience, including established procedures for navigating the safe transfer and storage of IPD.
- IPD meta-analyses of randomized clinical trials enhance the ability to handle participant- and study-level confounding, and increases the power to identify responder subgroups and mechanisms underlying treatment effects
- A key limitation to undertaking IPD analyses relates to overcoming data-sharing hurdles, and the achievement of our aims will in part depend on the ability to successfully obtain IPD from eligible studies.

For peer review only

Introduction

Knee osteoarthritis (OA) is a chronic musculoskeletal condition that affects approximately 24% of older adults (1). It is associated with substantial pain, loss of function, and reduced quality of life (2). There are currently no known disease-modifying treatment approaches available for knee OA. Current guidelines recommend a core approach of exercise, education and dietary weight management if appropriate (3). Adjunct interventions recommended for symptom management include pharmaceuticals such as non-steroidal anti-inflammatories, but also certain non-pharmacological interventions such as gait aids (3). At the end-stages of knee OA, when pain and disability become severe enough, knee arthroplasty is often undertaken. However, approximately 20% of individuals who undergo knee arthroplasty report not being satisfied following surgery (4-6). With no effective disease-modifying treatment options, individuals often spend decades living with pain and disability (7). It is thus clinically imperative to identify interventions that can contribute to symptom management in individuals living with knee OA.

Biomechanical factors, such as bony malalignment or poor movement patterns, may play a key role in the etiology of knee OA by contributing to abnormal forces across affected joints (8). Therefore, one broad class of interventions for knee OA involves application, or wear, of devices aimed at presumably improving an individual's biomechanics to reduce joint forces, improve symptoms, and potentially modify the disease trajectory. Such interventions include gait aids such as canes, but also bracing, taping, orthotics, and footwear, and they can easily serve as adjuncts to the current recommended core exercise-focused programs (3, 9, 10). This may be particularly relevant in individuals with certain biomechanical anomalies – such as frontal plane knee malalignment – because these individuals may be less likely to respond favourably to exercise (11). These commonly prescribed treatments are relatively inexpensive, less invasive, and have fewer side effects compared to other medical approaches such as intra-articular injections, oral medications, or surgery (12-14). Systematic reviews suggest some such

For peer review only - http://bmjopen.bmj.com/site/about/guidelines.xhtml

For peer review only

mechanical interventions (e.g., knee braces) may improve pain, though results have been conflicting or inconclusive regarding other outcomes or in other mechanical interventions (12-21).

One possible reason for conflicting results is that knee OA represents a heterogeneous disease, and certain subgroups with knee OA may respond better to mechanical interventions than others (22-24). For example, lateral wedge insoles and insoles with subtalar strapping may be more effective in individuals with varus knee alignment (13, 14, 17, 21), while medial wedge insoles may be more effective in individuals with valgus knee alignment (14). Including both of these groups of individuals in the same study could mask true treatment effects. While subgroup analyses within such a trial may successfully identify a 'responder' subgroup, most trials are not powered for such secondary analyses, and evaluating multiple possible subgroup characteristics further increases the likelihood of spurious findings (25-27). Confirming the existence of such 'responder' subgroups could lead to identifying effective targeted biomechanical interventions in knee OA.

In addition to subgroup characteristics, another possible reason for conflicting results is the mechanism by which such interventions impart their effect. If the effect of mechanical interventions is mediated by a change in some biomechanical feature, then it may be that different types (e.g., brace vs. tape), design, or dose of intervention will confer different outcomes via differing influence on the mediating feature (28). Confirming the mediating role that biomechanics may play in mechanical interventions could help to optimize the design and application of mechanical interventions.

Several systematic reviews have evaluated the efficacy of mechanical interventions in knee OA, however very little attention has been given to treatment effect modifiers or mediation analyses (12-21). Moreover, to our knowledge, no study on this topic has yet to pool individual participant data (IPD)

For peer review only - http://bmjopen.bmj.com/site/about/guidelines.xhtml

across studies. An IPD meta-analysis evaluates raw units of data rather than aggregate study-level data, and is thus a more robust approach to evaluating treatment effect modifiers and mediators (29, 30). Compared to traditional study-level meta-analyses, IPD meta-analyses of randomized control trials (RCTs) enhances the ability to handle participant- and study-level confounding, and increases the power to identify responder subgroups and mechanisms underlying treatment effects (30). The results using such an approach may therefore be more reliable and generalizable (30).

Despite the growing recognition of the ethical and scientific importance of data sharing and scientific transparency, one of the biggest challenges in undertaking IPD analyses relates to overcoming data-sharing hurdles (31-33). Barriers range from successfully reaching original study authors; willingness or ability of authors to share data; and international ethics and regulations issues (31-33). The OA Trial Bank is an internationally-recognized organization that was established in 2010 and has developed procedures for navigating these barriers, including safely sharing, handling and storing IPD data from RCTs (34). The OA Trial Bank steering committee supports and approves all projects, including providing input on research questions and study methods. This is therefore the ideal organization in the field of clinical OA research to collaborate with in successfully conducting an IPD meta-analysis.

Aims

We aim to conduct a systematic review with IPD meta-analysis of RCTs, under the guidance of the OA Trial Bank, to evaluate the efficacy of mechanical interventions (i.e., bracing, taping, orthoses, footwear, or canes) in managing knee OA symptoms. Our primary aim is to identify subgroups of individuals with knee OA who respond favourably to mechanical interventions. Our secondary aim is to evaluate the effect of biomechanics as a mediator between mechanical interventions and symptoms.

For peer review only - http://bmjopen.bmj.com/site/about/guidelines.xhtml

Methods and analyses

The OA Trial Bank steering committee approved a summary of this study protocol prior to preparing the full study protocol (34), and we published a basic study protocol with the International Prospective Register of Systematic Reviews (PROSPERO) (35). The current detailed protocol has been written in accordance with the Preferred Reporting Items for Systematic Reviews and Meta-Analysis Protocols (PRISMA-P) (36, 37) statement, and PRISMA Individual Participant Data (PRISMA-IPD) guidelines (29). In collaboration with the OA Trial Bank, we will use methods described previously to guide the transfer and use of IPD (38-40), updated recently to adhere to current European data-sharing regulations (41).

We will search for relevant studies in five databases: MEDLINE, Embase, CINAHL, CENTRAL, and Web of Science, with dates from inception to search date (21 August 2019). We will develop a search strategy in collaboration with an Erasmus MC librarian, using key words and medical subject headers (MeSH), and adapting the syntax to the respective indexing vocabularies of each database (see Appendix 1).

We will identify studies that meet the following eligibility criteria:

Participants: Adults (18 years or older) with knee OA (tibiofemoral or patellofemoral), diagnosed using any common method (e.g., radiographs, MRI, clinical criteria, diagnosis by a health care professional). We will include post-traumatic OA, and we will exclude patients who have undergone total knee arthroplasty or studies of patients with rheumatoid or other inflammatory arthropathies. If a study contains a subgroup of participants that meet our inclusion criteria, we will include that study if IPD data are retrieved, or if subgroup analyses are reported in the original publication.

For peer review only - http://bmjopen.bmj.com/site/about/guidelines.xhtml

Interventions: Any intervention involving use or wear of mechanical devices (e.g., bracing, taping, orthotics, footwear, cane) that is evaluated after more than one day or application of wear/use. We will include studies that combine these interventions with exercise or education/advice.

Comparison: Any non-surgical treatment (e.g. placebo, usual care, any other intervention that does not involve surgery), waiting list, or no treatment.

Outcomes: Our main outcome will be pain at the end of the study-specific primary duration of treatment. Treatment duration will be categorized as short-term (< 4 weeks), medium-term (4 – 12 weeks), and long-term (> 3 months). Outcome measures will also be extracted at additional time-points during treatment. We will not extract outcomes at any time-points that are measured after discontinuation of treatment. Secondary outcomes will include function, quality of life, global perceived change, OA feature severity, and biomechanics.

Study design: We will include peer-reviewed RCTs (or quasi-RCTs). We will exclude any other study design (e.g., non-RCTs, pre-post study designs, observational studies). We will also exclude RCTs that only measure the acute effects of a single application of treatment.

Languages: English, Dutch, German, French

Titles and abstracts will be initially imported into EndNote X9 (Clarivate Analytics) for deduplication, and then imported into Covidence for screening (42). Two independent reviewers (EMM, MJC) will screen titles and abstracts of all studies identified through this search strategy (43). A third reviewer (MvM) will be consulted in the event of unresolved disagreements. Following the initial screening, two reviewers

For peer review only - http://bmjopen.bmj.com/site/about/guidelines.xhtml

(EMM, MJC) will independently review full text manuscripts to identify studies for inclusion in this review. A third reviewer (MvM) will again be consulted in the event of disagreements. We will review reference lists of included studies and relevant reviews for additional eligible studies.

For all included studies (see Figure 1, flow chart), we will contact the corresponding author by e-mail. If a current e-mail address cannot be found or the author does not respond (up to three attempts), we will attempt to reach them by other means (e.g. phone, post, contact institution). Where IPD are available and authors or institutions are willing to share data, a data delivery agreement will be signed by both parties. Where local ethics regulations require it, ethics approval will be sought prior to sharing data. Pseudonymized or anonymized data sets (all formats are acceptable, e.g., SPSS, Excel) and related data dictionaries will then be transferred and stored securely on a database at Erasmus MC, for use only as agreed upon in the data delivery agreement. One original study investigator (first or senior author, at the discretion of the data owner) will be invited to be a co-author of the project if they are willing to assume responsibilities that meet authorship guidelines.

For peer review only

We will convert all data sets to a common format, combine data sets with a new variable identifying original trial, and harmonize variables. Data checking will include evaluating baseline characteristics and results of comparisons for our main outcomes against results reported in original publications. We will also check for balancing of baseline participant characteristics in each treatment arm, and evaluate the extent to which all randomized participants in the IPD datasets have been included in study analyses. Authors will be consulted in the case of any inconsistencies or discrepancies. In cases where discrepancies cannot be resolved, we will (on a case by case basis) either conduct a sensitivity analysis with that study removed, or we will exclude the study from our analysis altogether.

For peer review only - http://bmjopen.bmj.com/site/about/guidelines.xhtml

Data extraction:

Two independent investigators will extract data from all included published studies. From each study, we will extract the following data: country of study; funding source; study design; sample size; target population; inclusion/exclusion criteria; participant characteristics (age, sex, BMI, history of injury or surgery, comorbidities, psychosocial profile, metabolic profile, physical activity/fitness, lifestyle factors, medication use); type, dose and context of intervention (including compliance, co-interventions, protocol deviations, adverse events, drop-out or withdrawal details); OA characteristics (compartment involvement, prevalence, severity, tissues involved), including pre-post if available; and pain, function, quality of life, and biomechanics (e.g. proprioception, knee alignment, strength, kinematics, kinetics) pre-post as available. Global perceived change will also be extracted as available. Where IPD are available, we will conduct all analyses using IPD instead of aggregate data, following data consistency checks described above.

Risk of Bias:

Two co-authors will independently evaluate risk of bias (ROB) for each included study using the Cochrane ROB tool version 2 (44), and disagreements will be resolved by a third investigator. The Cochrane ROB v2 considers five domains of possible bias: randomization; deviations from intended interventions; missing outcome data; measurement of the outcome; and selection of the reported results. For each domain, ROB is rated as low, some concerns, or high. Both the original publications and IPD datasets will be used for evaluating ROB. The overall study will be considered to be of low ROB if all five domains are rated as low ROB, and high overall ROB if at least one domain is rated as high ROB or if some concerns are identified in multiple domains. We will consult original authors in the event of inadequate reporting or inconsistencies.

For peer review only - http://bmjopen.bmj.com/site/about/guidelines.xhtml

Statistical analyses:

We will conduct an IPD meta-analysis of short-term (<4 weeks), medium-term (4 – 12 weeks), and long-term (>3 months) effects of mechanical treatments in comparison to other non-surgical treatments, sham, waitlist, or usual care.

Where within-study missing data are sparse, we will assume data are missing completely at random and we will conduct complete case analyses, given the trivial loss of power and negligible implications on bias (45). Where higher proportions of data are missing within a study, we will conduct within-study multiple imputation (45, 46). In cases where a variable was not collected in a given study, we will exclude that study from the relevant analyses.

Treatment efficacy

To evaluate treatment efficacy, we will employ a two-step meta-analysis, first analysing each trial separately, and then pooling results across trials (30, 38, 47). In step one, within each trial, we will evaluate the effect of assigned intervention by intention to treat, regardless of method used in the original study. We anticipate that our main outcome, pain, will be evaluated differently across studies. To navigate this, we will evaluate the pain-related outcome from each study that ranks highest on the recommended hierarchy of pain-related outcomes to be used for meta-analyses (48) (see Table). For each available time point (short-, medium-, and long-term), we will fit an ANCOVA model to obtain the treatment effect estimate, including baseline pain as a covariate(49). We will report effect sizes from the ANCOVA model and its respective 95% confidence interval. In studies where we are unable to obtain IPD, we will extract aggregate data from published manuscripts as they are reported, for example based on final scores or change scores(50). Similar models will be performed for secondary outcomes as data

For peer review only - http://bmjopen.bmj.com/site/about/guidelines.xhtml

permit. In cases of dichotomous outcomes, we will perform binary modeling and report effect sizes as relative risk (RR, 95% CI).

Table. Hierarchy of pain-related outcomes proposed by Jüni et al 2006(48)

Rank	Pain outcome
Highest	Global pain score (e.g. NRS, VAS)
	Pain on walking (same as 1 but task-specific)
	WOMAC pain subscale(51)
	Other composite pain scores (e.g. KOOS Pain)(52)
	Pain on activities other than walking (e.g. stair climbing)
	WOMAC global score (all 3 subscales combined)
	Lequesne osteoarthritis index global score(53)
	Other algo-functional composite scores
	Patient's global assessment
Lowest	Physician's global assessment

NRS = numeric rating score; VAS = visual analogue scale; WOMAC = Western Ontario McMaster University Osteoarthritis Index; KOOS = Knee injury and Osteoarthritis Outcome Score

In step two, we will perform random effects meta-analysis employing restricted maximum likelihood (REML)(54). We will perform separate meta-analyses for each type of intervention (e.g., braces, taping) (30, 47, 55). We will report study heterogeneity as I^2 and τ^2 (56, 57). In cases of notable heterogeneity (I^2 > 50%)(57), we will consider possible sources such as device design, treatment duration, comparison treatment, or study quality. We will then consider performing meta-regression, subgroup analysis, or sensitivity analyses to explain or account for these potential sources of heterogeneity. We will pool results of studies both with and without IPD data after verifying that effect sizes of IPD studies do not differ from non-IPD studies (30, 50).

For peer review only - http://bmjopen.bmj.com/site/about/guidelines.xhtml

In addition to a two-step meta-analysis, we will also perform a one-step meta-analysis as a sensitivity analysis. In a one-step meta-analysis, all IPD datasets are harmonized into one large dataset and analysed together, with the addition of a covariate indicating original trial.

Treatment effect-modifier analyses

We will conduct treatment effect-modifier analyses to identify subgroups of individuals with knee OA who respond to various mechanical interventions (38). We have proposed several subgroup characteristics that we hypothesize may modify the effect of mechanical interventions on our main outcome, based on expert opinion. These proposed subgroups include the following baseline characteristics: (i) mild vs. severe OA (more severe joint space narrowing is associated with joint malalignment which may respond differently to mechanical interventions); (ii) tibiofemoral vs. patellofemoral OA (location of cartilage damage may be associated with differing joint alignment or source of symptoms); (iii) varus vs. valgus knee alignment (may be associated with different localized joint forces); (iv) obese vs. non-obese (may confer different amounts of mechanical stress); and (v) post-traumatic vs. non-traumatic OA (possible different biomechanical anomalies). Where feasible, we will apply a two-stage approach to investigate interactions using IPD data (27, 55). We will use a two-stage approach because it more easily separates within-trial and across-trial variation (27). We will conduct all treatment effect-modifier analyses in IPD data only (55).

Mediation analyses

We hypothesize that biomechanical factors may mediate the effect of these interventions (e.g., kinematics, kinetics, proprioception, hypermobility) by reducing or normalizing joint forces, which in turn reduces pain. If possible, we will conduct mediation analyses to evaluate this hypothesis (38). We acknowledge that it is rare for studies to evaluate biomechanical variables both before and after

For peer review only - http://bmjopen.bmj.com/site/about/guidelines.xhtml

treatment, so undertaking this analysis will depend on whether there are sufficient data available in included studies. If such an analysis is possible, we will employ a single mediator model to evaluate the proportion of the total effect of the intervention on pain that is mediated by a change in biomechanics (58).

We will conduct funnel plot analyses where there are at least ten studies for a given intervention, to consider the possible effects of small sample size or publication bias (59). We will summarize the overall level of evidence for each category of intervention using the GRADE approach (60).

All analyses will be performed using Stata 15.1 (StataCorp, USA).

Patient and public involvement

Patients and the public were not directly involved in the design of this study protocol. The OA Trial Bank advisory board includes patient-members who provide overall input to the OA Trial Bank Steering Committee activities. We will solicit patient involvement through the OA Trial Bank advisory board and also through our institutional patient advisors (www.arthrosegezond.nl) for input in the analysis and interpretation of our study results, and to inform and guide dissemination of our study results.

Ethics and dissemination

No new data will be collected, so de novo ethics approval is not required for our study. The OA Trial Bank has established protocols in place to guide the confidential and secure transfer and use of pseudonymized IPD (38-40) that adheres to current European data-sharing regulations (41). We will collaborate with data deliverers to also adhere to relevant institutional, national or international regulations regarding data sharing and ethics. We will store all IPD datasets on a secure driver in

For peer review only - http://bmjopen.bmj.com/site/about/guidelines.xhtml

accordance with OA Trial Bank procedures. We will disseminate findings via international conferences, open-source publication in peer-reviewed journals, and summaries posted on websites serving the public and clinicians.

Acknowledgements

We thank Dr. Wichor Bramer at Erasmus MC for assisting us with developing our search terms and managing our search.

Author contributions

All authors contributed to the concept, design, and methodology of this study, and have provided substantial intellectually input to this manuscript, including approval of the final manuscript.

Funding

Dr. Macri is funded by a Canadian Institutes of Health Research (CIHR) Banting Postdoctoral Fellowship. The OA Trial Bank has received funding from ReumaNederland.

Competing interests

SMABZ reports grants from The Netherlands Organisation for Health Research and Development, CZ, European Union, Foreum, Dutch Arthritis Association, personal fees from Osteoarthritis Research Society International (OARSI), personal fees from Infirst Healthcare, and personal fees from Phizer, all outside of the submitted work. We have no other disclosures.

For peer review only - http://bmjopen.bmj.com/site/about/guidelines.xhtml

References

1. Pereira D, Peleteiro B, Araujo J, Branco J, Santos RA, Ramos E. The effect of osteoarthritis definition on prevalence and incidence estimates: a systematic review. Osteoarthritis Cartilage. 2011;19(11):1270-85.

2. Guccione AA, Felson DT, Anderson JJ, Anthony JM, Zhang Y, Wilson PW, et al. The effects of specific medical conditions on the functional limitations of elders in the Framingham Study. Am J Public Health. 1994;84(3):351-8.

3. Bannuru RR, Osani MC, Vaysbrot EE, Arden NK, Bennell K, Bierma-Zeinstra SMA, et al. OARSI guidelines for the non-surgical management of knee, hip, and polyarticular osteoarthritis. Osteoarthritis Cartilage. 2019;27(11):1578-89.

4. Beswick AD, Wylde V, Gooberman-Hill R, Blom A, Dieppe P. What proportion of patients report long-term pain after total hip or knee replacement for osteoarthritis? A systematic review of prospective studies in unselected patients. BMJ Open. 2012;2(1):e000435.

5. Hawker GA, Badley EM, Croxford R, Coyte PC, Glazier RH, Guan J, et al. A population-based nested case-control study of the costs of hip and knee replacement surgery. Med Care. 2009:732-41.

6. Robertsson O, Dunbar M, Pehrsson T, Knutson K, Lidgren L. Patient satisfaction after knee arthroplasty: a report on 27,372 knees operated on between 1981 and 1995 in Sweden. Acta Orthopaedica Scandinavica. 2000;71(3):262-7.

7. Ackerman IN, Kemp JL, Crossley KM, Culvenor AG, Hinman RS. Hip and Knee Osteoarthritis Affects Younger People, Too. J Orthop Sports Phys Ther. 2017;47(2):67-79.

8. Felson DT. Osteoarthritis as a disease of mechanics. Osteoarthritis Cartilage. 2013;21(1):10-5.

9. Crossley KM, Vicenzino B, Lentzos J, Schache AG, Pandy MG, Ozturk H, et al. Exercise, education, manual-therapy and taping compared to education for patellofemoral osteoarthritis: A blinded, randomised clinical trial. Osteoarthritis Cartilage. 2015;23(9):1457-64.

For peer review only

10. Skou ST, Roos EM, Laursen MB, Rathleff MS, Arendt-Nielsen L, Rasmussen S, et al. Total knee replacement and non-surgical treatment of knee osteoarthritis: 2-year outcome from two parallel randomized controlled trials. Osteoarthritis Cartilage. 2018;26(9):1170-80.

11. Quicke JG, Runhaar J, van der Windt D, Healey EL, Foster NE, Holden MA. Moderators of the effects of exercise for hip and knee osteoarthritis: a systematic review of subgroup analyses from randomised controlled trials. Osteoarthritis Cartilage. 2020;28:S161.

12. Gohal C, Shanmugaraj A, Tate P, Horner NS, Bedi A, Adili A, et al. Effectiveness of valgus offloading knee braces in the treatment of medial compartment knee osteoarthritis: a systematic review. Sports health. 2018;10(6):500-14.

13. Penny P, Geere J, Smith TO. A systematic review investigating the efficacy of laterally wedged insoles for medial knee osteoarthritis. Rheumatology international. 2013;33(10):2529-38.

14. Raja K, Dewan N. Efficacy of knee braces and foot orthoses in conservative management of knee osteoarthritis: a systematic review. Am J Phys Med Rehabil. 2011;90(3):247-62.

15. Mine K, Nakayama T, Milanese S, Grimmer K. The effectiveness of braces and orthoses for patients with knee osteoarthritis: A systematic review of Japanese-language randomised controlled trials. Prosthetics and orthotics international. 2017;41(2):115-26.

16. Cherian JJ, Jauregui JJ, Leichliter AK, Elmallah RK, Bhave A, Mont MA. The effects of various physical non-operative modalities on the pain in osteoarthritis of the knee. The bone & joint journal. 2016;98(1_Supple_A):89-94.

17. Brouwer RW, Jakma TS, Verhagen AP, Verhaar JA, Bierma-Zeinstra SM. Braces and orthoses for treating osteoarthritis of the knee. Cochrane Database Syst Rev. 2005(1):CD004020.

18. Cudejko T, van der Esch M, van der Leeden M, Roorda L, Pallari J, Bennell K, et al. Effect of soft braces on pain and physical function in patients with knee osteoarthritis: systematic review with meta-analyses. Osteoarthritis Cartilage. 2017;25:S390-S1.

For peer review only

For peer review only - http://bmjopen.bmj.com/site/about/guidelines.xhtml

19. Ouyang J-H, Chang K-H, Hsu W-Y, Cho Y-T, Liou T-H, Lin Y-N. Non-elastic taping, but not elastic taping, provides benefits for patients with knee osteoarthritis: systemic review and meta-analysis. Clinical rehabilitation. 2018;32(1):3-17.

20. Lu Z, Li X, Chen R, Guo C. Kinesio taping improves pain and function in patients with knee osteoarthritis: A meta-analysis of randomized controlled trials. International Journal of Surgery. 2018.

21. Reilly KA, Barker KL, Shamley D. A systematic review of lateral wedge orthotics—how useful are they in the management of medial compartment osteoarthritis? The Knee. 2006;13(3):177-83.

22. Deveza LA, Loeser RF. Is osteoarthritis one disease or a collection of many? Rheumatology. 2018;57(suppl_4):iv34-iv42.

23. Loeser RF, Goldring SR, Scanzello CR, Goldring MB. Osteoarthritis: a disease of the joint as an organ. Arthritis Rheum. 2012;64(6):1697-707.

24. Bierma-Zeinstra S, Verhagen AP. Osteoarthritis subpopulations and implications for clinical trial design. Arthritis Res Ther. 2011;13:213.

25. Sun X, Ioannidis JPA, Agoritsas T, Alba AC, Guyatt G. How to use a subgroup analysis: users' guide to the medical literature. Jama. 2014;311(4):405-11.

26. Hancock M, Herbert RD, Maher CG. A guide to interpretation of studies investigating subgroups of responders to physical therapy interventions. Physical therapy. 2009;89(7):698-704.

27. Fisher DJ, Carpenter JR, Morris TP, Freeman SC, Tierney JF. Meta-analytical methods to identify who benefits most from treatments: daft, deluded, or deft approach? bmj. 2017;356:j573.

28. Kraemer HC. Messages for clinicians: moderators and mediators of treatment outcome in randomized clinical trials. Am J Psychiatry. 2016;173(7):672-9.

29. Stewart LA, Clarke M, Rovers M, Riley RD, Simmonds M, Stewart G, et al. Preferred reporting items for a systematic review and meta-analysis of individual participant data: the PRISMA-IPD statement. Jama. 2015;313(16):1657-65.

For peer review only

30. Riley RD, Lambert PC, Abo-Zaid G. Meta-analysis of individual participant data: rationale, conduct, and reporting. Bmj. 2010;340:c221.

31. van Middelkoop M, Lohmander S, Bierma-Zeinstra SMA. Sharing data–taming the beast: barriers to meta-analyses of individual patient data (IPD) and solutions. BMJ Publishing Group Ltd and British Association of Sport and Exercise Medicine; 2020.

32. Lo B. Sharing clinical trial data: maximizing benefits, minimizing risk. Jama. 2015;313(8):793-4.

33. Packer M. Data sharing in medical research. British Medical Journal Publishing Group; 2018.

34. The OA Trial Bank. Individual patient data meta-analysis in osteoarthritis research. [cited 2020 29 May]. Available from: http://www.oatrialbank.com/home.

35. PROSPERO International Prospective Register of Systematic Reviews [cited 2014]. Available from: http://www.crd.york.ac.uk/prospero.

36. Moher D, Shamseer L, Clarke M, Ghersi D, Liberati A, Petticrew M, et al. Preferred reporting items for systematic review and meta-analysis protocols (PRISMA-P) 2015 statement. Systematic reviews. 2015;4(1):1.

37. Shamseer L, Moher D, Clarke M, Ghersi D, Liberati A, Petticrew M, et al. Preferred reporting items for systematic review and meta-analysis protocols (PRISMA-P) 2015: elaboration and explanation. Bmj. 2015;349:g7647.

38. Holden MA, Burke DL, Runhaar J, van Der Windt D, Riley RD, Dziedzic K, et al. Subgrouping and TargetEd Exercise pRogrammes for knee and hip OsteoArthritis (STEER OA): a systematic review update and individual participant data meta-analysis protocol. BMJ open. 2017;7(12):e018971.

39. Deveza LA, Bierma-Zeinstra SMA, Van Spil WE, Oo WM, Saragiotto BT, Neogi T, et al. Efficacy of bisphosphonates in specific knee osteoarthritis subpopulations: protocol for an OA Trial Bank systematic review and individual patient data meta-analysis. BMJ open. 2018;8(12).

For peer review only
For peer review only - http://bmjopen.bmj.com/site/about/guidelines.xhtml

40. van Middelkoop M, Arden NK, Atchia I, Birrell F, Chao J, Rezende MU, et al. The OA Trial Bank: meta-analysis of individual patient data from knee and hip osteoarthritis trials show that patients with severe pain exhibit greater benefit from intra-articular glucocorticoids. Osteoarthritis Cartilage. 2016;24(7):1143-52.

41. Union EPatCotE. General Data Protection Regulation [29 May 2020]. Available from: https://gdpr-info.eu/.

42. Covidence Systematic Review Software Veritas Health Innovation, Melbourne, Australia. Available from: www.covidence.org.

43. Bramer WM, Milic J, Mast F. Reviewing retrieved references for inclusion in systematic reviews using EndNote. Journal of the Medical Library Association: JMLA. 2017;105(1):84.

44. Higgins JPT, Sterne JAC, Savović J, Page MJ, Hrobjartsson A, Boutron I, et al. A revised tool for assessing risk of bias in randomized trials. Chander J, McKenzie J, Boutron I, Welch V, editors: Cochrane Database of Systematic Reviews; 2016.

45. Quartagno M, Carpenter JR. Multiple imputation for IPD meta-analysis: allowing for heterogeneity and studies with missing covariates. Stat Med. 2016;35(17):2938-54.

46. Koopman L, van der Heijden GJMG, Grobbee DE, Rovers MM. Comparison of methods of handling missing data in individual patient data meta-analyses: an empirical example on antibiotics in children with acute otitis media. Am J Epidemiol. 2008;167(5):540-5.

47. Riley RD, Lambert PC, Staessen JA, Wang J, Gueyffier F, Thijs L, et al. Meta-analysis of continuous outcomes combining individual patient data and aggregate data. Stat Med. 2008;27(11):1870-93.

48. Jüni P, Reichenbach S, Dieppe P. Osteoarthritis: rational approach to treating the individual. Best practice & research Clinical rheumatology. 2006;20(4):721-40.

For peer review only - http://bmjopen.bmj.com/site/about/guidelines.xhtml

49. Zhang S, Paul J, Nantha-Aree M, Buckley N, Shahzad U, Cheng J, et al. Empirical comparison of four baseline covariate adjustment methods in analysis of continuous outcomes in randomized controlled trials. Clin Epidemiol. 2014;6:227.

50. Higgins J, Thomas J, Chandler J, Cumpston M, Li T, Page M, et al., editors. Cochrane Handbook for Systematic Reviews of Interventions version 6.0 (updated July 2019): Cochrane; 2019.

51. Bellamy N. WOMAC Osteoarthritis Index. A user's guide. London: University of Western Ontario; 1995.

52. Roos EM, Roos HP, Lohmander LS, Ekdahl C, Beynnon BD. Knee Injury and Osteoarthritis Outcome Score (KOOS)--development of a self-administered outcome measure. J Orthop Sports Phys Ther. 1998;28(2):88-96.

53. Lequesne MG, Mery C, Samson M, Gerard P. Indexes of severity for osteoarthritis of the hip and knee: validation–value in comparison with other assessment tests. Scand J Rheumatol. 1987;16(sup65):85-9.

54. Langan D, Higgins JPT, Jackson D, Bowden J, Veroniki AA, Kontopantelis E, et al. A comparison of heterogeneity variance estimators in simulated random-effects meta-analyses. Research synthesis methods. 2019;10(1):83-98.

55. Fisher DJ. Two-stage individual participant data meta-analysis and generalized forest plots. The Stata Journal. 2015;15(2):369-96.

56. Higgins JPT, Thompson SG, Deeks JJ, Altman DG. Measuring inconsistency in meta-analyses. BMJ. 2003;327(7414):557-60.

57. Higgins JPT, Thompson SG. Quantifying heterogeneity in a meta-analysis. Stat Med. 2002;21(11):1539-58.

58. Lee H, Herbert RD, Lamb SE, Moseley AM, McAuley JH. Investigating causal mechanisms in randomised controlled trials. Trials. 2019;20(1):1-5.

For peer review only

For peer review only - http://bmjopen.bmj.com/site/about/guidelines.xhtml

59. Sterne JAC, Sutton AJ, Ioannidis JPA, Terrin N, Jones DR, Lau J, et al. Recommendations for examining and interpreting funnel plot asymmetry in meta-analyses of randomised controlled trials. Bmj. 2011;343:d4002.

60. Atkins D, Eccles M, Flottorp S, Guyatt GH, Henry D, Hill S, et al. Systems for grading the quality of evidence and the strength of recommendations I: critical appraisal of existing approaches The GRADE Working Group. BMC Health Serv Res. 2004;4(1):38.

For peer review only

For peer review only - http://bmjopen.bmj.com/site/about/guidelines.xhtml

Appendix 1. Search Terms

Medline (OVID)

(Osteoarthritis, Knee/ OR ((Knee Joint/ OR Knee/ OR Patellofemoral Joint/ OR knee function/) AND Osteoarthritis/) OR (((knee* OR femorotib* OR tibiofemor* OR patellofemoral* OR femoropatell* OR gon) ADJ6 (osteoarth* OR arthrit* OR arthrosis OR oa)) OR (femor* ADJ3 (tibi* OR patell*) ADJ6 (osteoarth* OR arthrit* OR arthrosis OR oa)) OR gonarthr*).ab,ti.) AND (Orthotic Devices/ OR Braces/ OR Foot Orthoses/ OR Shoes/ OR Canes/ OR Crutches/ OR Athletic Tape/ OR (orthos* OR orthotic* OR brace* OR bracing* OR ((orthop* OR foot-worn) ADJ3 (support* OR device* OR applian* OR apparat* OR platform*)) OR shoe* OR crutch* OR cane OR canes OR tape OR taping OR kinesiotap* OR KneeBrac* OR ((mechanical* OR biomechan*) ADJ3 (intervent* OR treat* OR therap*)) OR insole* OR sleeve* OR footwear* OR foot-wear* OR walking-stick* OR walking-aid* OR ((elastic* OR adhesi*) ADJ3 bandage*)).ab,ti.) AND (Exp Controlled clinical trial/ OR "Double-Blind Method"/ OR "Single-Blind Method"/ OR "Random Allocation"/ OR (random* OR factorial* OR crossover* OR cross over* OR placebo* OR ((doubl* OR singl*) ADJ blind*) OR assign* OR allocat* OR volunteer* OR trial OR groups).ab,ti.) NOT (exp Animals/ NOT Humans/)

embase.com

('knee osteoarthritis'/de OR 'patellofemoral arthritis'/de OR (('knee pain'/de OR 'knee'/de OR 'patellofemoral joint'/de OR 'knee function'/de) AND osteoarthritis/de) OR 'Knee Injury and Osteoarthritis Outcome'/de OR 'knee arthritis'/de OR (((knee* OR femorotib* OR tibiofemor* OR patellofemoral* OR femoropatell* OR gon) NEAR/6 (osteoarth* OR arthrit* OR arthrosis OR oa)) OR (femor* NEAR/3 (tibi* OR patell*) NEAR/6 (osteoarth* OR arthrit* OR arthrosis OR oa)) OR gonarthr*):ab,ti) AND ('orthosis'/de OR orthotics/de OR brace/de OR 'knee orthosis'/exp OR 'inshoe orthosis'/de OR 'leg orthosis'/de OR 'foot orthosis'/exp OR 'walking orthosis'/de OR 'shoe'/de OR 'cane'/de OR crutch/exp OR 'walking aid'/exp OR 'orthopedic shoe'/exp OR 'athletic tape'/de OR 'kinesiotape'/de OR 'kinesio tape'/de OR 'kinesio taping'/de OR 'kinesiotaping'/de OR 'bandaging technique'/de OR 'elastic adhesive bandage'/de OR (orthos* OR orthotic* OR brace* OR bracing* OR ((orthop* OR foot-worn) NEAR/3 (support* OR device* OR applian* OR apparat* OR platform*)) OR shoe* OR crutch* OR cane OR canes OR tape OR taping OR kinesiotap* OR KneeBrac* OR ((mechanical* OR biomechan*) NEAR/3 (intervent* OR treat* OR therap*)) OR insole* OR sleeve* OR footwear* OR foot-wear* OR walking-stick* OR walking-aid* OR ((elastic* OR adhesi*) NEAR/3 bandage*)):ab,ti) AND ('Controlled clinical trial'/exp OR 'Crossover procedure'/de OR 'Double-blind procedure'/de OR 'Single-blind procedure'/de OR (random* OR factorial* OR crossover* OR (cross NEXT/1 over*) OR placebo* OR ((doubl* OR singl*) NEXT/1 blind*) OR assign* OR allocat* OR volunteer* OR trial OR groups):ab,ti) NOT ([animals]/lim NOT [humans]/lim)

CINAHL EBSCOhost

For peer review only

(Osteoarthritis, Knee+ OR ((Knee Joint+ OR Knee+ OR Patellofemoral Joint+ OR knee function+) AND Osteoarthritis+) OR (((knee* OR femorotib* OR tibiofemor* OR patellofemoral* OR femoropatell* OR gon) N5 (osteoarth* OR arthrit* OR arthrosis OR oa)) OR (femor* N2 (tibi* OR patell*) N5 (osteoarth* OR arthrit* OR arthrosis OR oa)) OR gonarthr*)) AND (Orthotic Devices+ OR Braces+ OR Foot Orthoses+ OR Shoes+ OR Canes+ OR Crutches+ OR Athletic Tape+ OR (orthos* OR orthotic* OR brace* OR bracing* OR ((orthop* OR foot-worn) N2 (support* OR device* OR applian* OR apparat* OR platform*)) OR shoe* OR crutch* OR cane OR canes OR tape OR taping OR kinesiotap* OR KneeBrac* OR ((mechanical* OR biomechan*) N2 (intervent* OR treat* OR therap*)) OR insole* OR sleeve* OR footwear* OR foot-wear* OR walking-stick* OR walking-aid* OR ((elastic* OR adhesi*) N2 bandage*))) AND (MH Controlled clinical trial+ OR "Double-Blind Method+" OR "Single-Blind Method+" OR "Random Allocation+" OR (random* OR factorial* OR crossover* OR cross over* OR placebo* OR ((doubl* OR singl*) N1 blind*) OR assign* OR allocat* OR volunteer* OR trial OR groups)) NOT (MH Animals+ NOT Humans+)

Cochrane CENTRAL registry of trials

((((knee* OR femorotib* OR tibiofemor* OR patellofemoral* OR femoropatell* OR gon) NEAR/6 (osteoarth* OR arthrit* OR arthrosis OR oa)) OR (femor* NEAR/3 (tibi* OR patell*) NEAR/6 (osteoarth* OR arthrit* OR arthrosis OR oa)) OR gonarthr*):ab,ti) AND ((orthos* OR orthotic* OR brace* OR bracing* OR ((orthop* OR foot next worn) NEAR/3 (support* OR device* OR applian* OR apparat* OR platform*)) OR shoe* OR crutch* OR cane OR canes OR tape OR taping OR kinesiotap* OR KneeBrac* OR ((mechanical* OR biomechan*) NEAR/3 (intervent* OR treat* OR therap*)) OR insole* OR sleeve* OR footwear* OR foot next wear* OR walking next stick* OR walking next aid* OR ((elastic* OR adhesi*) NEAR/3 bandage*)):ab,ti)

Web of science Core Collection

TS=(((((knee* OR femorotib* OR tibiofemor* OR patellofemoral* OR femoropatell* OR gon) NEAR/5 (osteoarth* OR arthrit* OR arthrosis OR oa)) OR (femor* NEAR/2 (tibi* OR patell*) NEAR/5 (osteoarth* OR arthrit* OR arthrosis OR oa)) OR gonarthr*)) AND ((orthos* OR orthotic* OR brace* OR bracing* OR ((orthop* OR foot-worn) NEAR/2 (support* OR device* OR applian* OR apparat* OR platform*)) OR shoe* OR crutch* OR cane OR canes OR tape OR taping OR kinesiotap* OR KneeBrac* OR ((mechanical* OR biomechan*) NEAR/2 (intervent* OR treat* OR therap*)) OR insole* OR sleeve* OR footwear* OR foot-wear* OR walking-stick* OR walking-aid* OR ((elastic* OR adhesi*) NEAR/2 bandage*))) AND (random* OR factorial* OR crossover* OR cross over* OR placebo* OR ((doubl* OR singl*) N1 blind*) OR assign* OR allocat* OR volunteer* OR trial OR groups))

For peer review only

PRISMA-P (Preferred Reporting Items for Systematic review and Meta-Analysis Protocols) 2015 checklist: recommended items to address in a systematic review protocol*

Section and topic	Item No	Checklist item	Page Reported
**ADMINISTRATIVE INFORMATION**			
Title:			
Identification	1a	Identify the report as a protocol of a systematic review	1
Update	1b	If the protocol is for an update of a previous systematic review, identify as such	
Registration	2	If registered, provide the name of the registry (such as PROSPERO) and registration number	2
Authors:			
Contact	3a	Provide name, institutional affiliation, e-mail address of all protocol authors; provide physical mailing address of corresponding author	1
Contributions	3b	Describe contributions of protocol authors and identify the guarantor of the review	15
Amendments	4	If the protocol represents an amendment of a previously completed or published protocol, identify as such and list changes; otherwise, state plan for documenting important protocol amendments	n/a
Support:			
Sources	5a	Indicate sources of financial or other support for the review	15
Sponsor	5b	Provide name for the review funder and/or sponsor	
Role of sponsor or funder	5c	Describe roles of funder(s), sponsor(s), and/or institution(s), if any, in developing the protocol	
**INTRODUCTION**			
Rationale	6	Describe the rationale for the review in the context of what is already known	4-6
Objectives	7	Provide an explicit statement of the question(s) the review will address with reference to participants, interventions, comparators, and outcomes (PICO)	6
**METHODS**			
Eligibility criteria	8	Specify the study characteristics (such as PICO, study design, setting, time frame) and report characteristics (such as years considered, language, publication status) to be used as criteria for eligibility for the review	7-8
Information sources	9	Describe all intended information sources (such as electronic databases, contact with study authors, trial registers or other grey literature sources) with planned dates of coverage	7
Search strategy	10	Present draft of search strategy to be used for at least one electronic database, including planned limits, such that it could be repeated	App 1
Study records:			

For peer review only

Data management	11a	Describe the mechanism(s) that will be used to manage records and data throughout the review	
Selection process	11b	State the process that will be used for selecting studies (such as two independent reviewers) through each phase of the review (that is, screening, eligibility and inclusion in meta-analysis)	8-10
Data collection process	11c	Describe planned method of extracting data from reports (such as piloting forms, done independently, in duplicate), any processes for obtaining and confirming data from investigators	
Data items	12	List and define all variables for which data will be sought (such as PICO items, funding sources), any pre-planned data assumptions and simplifications	
Outcomes and prioritization	13	List and define all outcomes for which data will be sought, including prioritization of main and additional outcomes, with rationale	8,13
Risk of bias in individual studies	14	Describe anticipated methods for assessing risk of bias of individual studies, including whether this will be done at the outcome or study level, or both; state how this information will be used in data synthesis	10
Data synthesis	15a	Describe criteria under which study data will be quantitatively synthesised	9,11-14
	15b	If data are appropriate for quantitative synthesis, describe planned summary measures, methods of handling data and methods of combining data from studies, including any planned exploration of consistency (such as I^2, Kendall's τ)	
	15c	Describe any proposed additional analyses (such as sensitivity or subgroup analyses, meta-regression)	
	15d	If quantitative synthesis is not appropriate, describe the type of summary planned	
Meta-bias(es)	16	Specify any planned assessment of meta-bias(es) (such as publication bias across studies, selective reporting within studies)	14
Confidence in cumulative evidence	17	Describe how the strength of the body of evidence will be assessed (such as GRADE)	14

*** It is strongly recommended that this checklist be read in conjunction with the PRISMA-P Explanation and Elaboration (cite when available) for important clarification on the items. Amendments to a review protocol should be tracked and dated. The copyright for PRISMA-P (including checklist) is held by the PRISMA-P Group and is distributed under a Creative Commons Attribution Licence 4.0.**

From: Shamseer L, Moher D, Clarke M, Ghersi D, Liberati A, Petticrew M, Shekelle P, Stewart L, PRISMA-P Group. Preferred reporting items for systematic review and meta-analysis protocols (PRISMA-P) 2015: elaboration and explanation. BMJ. 2015 Jan 2;349(jan02 1):g7647.

For peer review only

BMJ Open

The effects of mechanical interventions in the management of knee osteoarthritis: protocol for an OA Trial Bank systematic review and individual participant data meta-analysis

Journal:	*BMJ Open*
Manuscript ID	bmjopen-2020-043026.R1
Article Type:	Protocol
Date Submitted by the Author:	21-Dec-2020
Complete List of Authors:	Macri, Erin; Erasmus MC, Orthopaedics and Sport Medicine; The University of British Columbia, Family Practice<br>Callaghan, Michael; Manchester Metropolitan University, Health Professions<br>van Middelkoop, Marienke; Erasmus MC, General Practice<br>Hattle, Miriam; Keele University<br>Bierma-Zeinstra, Sita; Erasmus University Medical Centre, Department of General Practice
**Primary Subject Heading**:	Rehabilitation medicine
Secondary Subject Heading:	Evidence based practice
Keywords:	Knee < ORTHOPAEDIC & TRAUMA SURGERY, RHEUMATOLOGY, REHABILITATION MEDICINE

SCHOLARONE™
Manuscripts

I, the Submitting Author has the right to grant and does grant on behalf of all authors of the Work (as defined in the below author licence), an exclusive licence and/or a non-exclusive licence for contributions from authors who are: i) UK Crown employees; ii) where BMJ has agreed a CC-BY licence shall apply, and/or iii) in accordance with the terms applicable for US Federal Government officers or employees acting as part of their official duties; on a worldwide, perpetual, irrevocable, royalty-free basis to BMJ Publishing Group Ltd ("BMJ") its licensees and where the relevant Journal is co-owned by BMJ to the co-owners of the Journal, to publish the Work in this journal and any other BMJ products and to exploit all rights, as set out in our licence.

The Submitting Author accepts and understands that any supply made under these terms is made by BMJ to the Submitting Author unless you are acting as an employee on behalf of your employer or a postgraduate student of an affiliated institution which is paying any applicable article publishing charge ("APC") for Open Access articles. Where the Submitting Author wishes to make the Work available on an Open Access basis (and intends to pay the relevant APC), the terms of reuse of such Open Access shall be governed by a Creative Commons licence – details of these licences and which Creative Commons licence will apply to this Work are set out in our licence referred to above.

Other than as permitted in any relevant BMJ Author's Self Archiving Policies, I confirm this Work has not been accepted for publication elsewhere, is not being considered for publication elsewhere and does not duplicate material already published. I confirm all authors consent to publication of this Work and authorise the granting of this licence.

For peer review only

The effects of mechanical interventions in the management of knee osteoarthritis: protocol for an OA Trial Bank systematic review and individual participant data meta-analysis

Erin M Macri[1] e.macri@erasmusmc.nl
Michael J Callaghan[2] michael.callaghan@mmu.ac.uk
Marienke van Middelkoop[1] m.vanmiddelkoop@erasmusmc.nl
Miriam Hattle[3] m.s.hattle@keele.ac.uk
Sita MA Bierma-Zeinstra[1] s.bierma-zeinstra@erasmusmc.nl

Affiliations: 1. Erasmus MC, Rotterdam, NL; 2. Manchester Metropolitan University, Manchester, UK; 3. Keele University, Staffordshire, UK

Correspondance:
EM Macri
Erasmus University Medical Center Rotterdam
Na1907, Wytemaweg 80, 3015 CN Rotterdam, The Netherlands

For peer review only - http://bmjopen.bmj.com/site/about/guidelines.xhtml

Abstract

Introduction: Knee osteoarthritis (OA) is a prevalent and disabling musculoskeletal condition. Biomechanical factors may play a key role in the etiology of knee OA, therefore a broad class of interventions involves the application or wear of devices designed to mechanically support knees with OA. These include gait aids, bracing, taping, orthotics, and footwear. The literature regarding efficacy of mechanical interventions has been conflicting or inconclusive, and this may be because certain subgroups with knee OA respond better to mechanical interventions. Our primary aim is to identify subgroups with knee OA who respond favourably to mechanical interventions. *Methods and analysis:* We will conduct a systematic review to identify randomized clinical trials of any mechanical intervention for the treatment of knee OA. We will invite lead authors of eligible studies to share individual participant data (IPD). We will perform an IPD meta-analysis for each type of mechanical intervention to evaluate efficacy, with our main outcome being pain. Where IPD are not available, this will be achieved using aggregate data. We will then evaluate five potential treatment effect-modifiers using a two-stage approach. If data permit, we will also evaluate whether biomechanics mediate the effects of mechanical interventions on pain in knee OA.

Ethics and dissemination: No new data will be collected in this study. We will adhere to institutional, national and international regulations regarding the secure and confidential sharing of IPD, addressing ethics as indicated. We will disseminate findings via international conferences, open-source publication in peer-reviewed journals, and summaries posted on websites serving the public and clinicians.

Registration: PROSPERO CRD42020155466

For peer review only - http://bmjopen.bmj.com/site/about/guidelines.xhtml

Strengths and limitations of this study

- We designed our protocol in collaboration with the OA Trial Bank, an internationally-recognized organization with considerable IPD experience, including established procedures for navigating the safe transfer and storage of IPD.
- IPD meta-analyses of randomized clinical trials enhance the ability to handle participant- and study-level confounding, and increases the power to identify responder subgroups and mechanisms underlying treatment effects
- A key limitation to undertaking IPD analyses relates to overcoming data-sharing hurdles, and the achievement of our aims will in part depend on the ability to successfully obtain IPD from eligible studies.

For peer review only - http://bmjopen.bmj.com/site/about/guidelines.xhtml

Introduction

Knee osteoarthritis (OA) is a chronic musculoskeletal condition that affects approximately 24% of older adults (1). It is associated with substantial pain, loss of function, and reduced quality of life (2). There are currently no known disease-modifying treatment approaches available for knee OA. Current guidelines recommend a core approach of exercise, education and dietary weight management if appropriate (3). Adjunct interventions recommended for symptom management include pharmaceuticals such as non-steroidal anti-inflammatories, but also certain non-pharmacological interventions such as gait aids (3). At the end-stages of knee OA, when pain and disability become severe enough, knee arthroplasty is often undertaken. However, approximately 20% of individuals who undergo knee arthroplasty report not being satisfied following surgery (4-6). With no effective disease-modifying treatment options, individuals often spend decades living with pain and disability (7). It is thus clinically imperative to identify interventions that can contribute to symptom management in individuals living with knee OA.

Biomechanical factors, such as bony malalignment or poor movement patterns, may play a key role in the etiology of knee OA by contributing to abnormal forces across affected joints (8). Therefore, one broad class of interventions for knee OA involves application, or wear, of devices aimed at presumably improving an individual's biomechanics to reduce joint forces, improve symptoms, and potentially modify the disease trajectory. Such interventions include gait aids such as canes, but also bracing, taping, orthotics, and footwear, and they can easily serve as adjuncts to the current recommended core exercise-focused programs (3, 9, 10). This may be particularly relevant in individuals with certain biomechanical anomalies – such as frontal plane knee malalignment – because these individuals may be less likely to respond favourably to exercise (11). These commonly prescribed treatments are relatively inexpensive, less invasive, and have fewer side effects compared to other medical approaches such as intra-articular injections, oral medications, or surgery (12-14). Systematic reviews suggest some such

For peer review only - http://bmjopen.bmj.com/site/about/guidelines.xhtml

mechanical interventions (e.g., knee braces) may improve pain, though results have been conflicting or inconclusive regarding other outcomes or in other mechanical interventions (12-21).

One possible reason for conflicting results is that knee OA represents a heterogeneous disease, and certain subgroups with knee OA may respond better to mechanical interventions than others (22-24). For example, lateral wedge insoles and insoles with subtalar strapping may be more effective in individuals with varus knee alignment (13, 14, 17, 21), while medial wedge insoles may be more effective in individuals with valgus knee alignment (14). Including both of these groups of individuals in the same study could mask true treatment effects. While subgroup analyses within such a trial may successfully identify a ‘responder’ subgroup, most trials are not powered for such secondary analyses, and evaluating multiple possible subgroup characteristics further increases the likelihood of spurious findings (25-27). Confirming the existence of such ‘responder’ subgroups could lead to identifying effective targeted biomechanical interventions in knee OA.

In addition to subgroup characteristics, another possible reason for conflicting results is the mechanism by which such interventions impart their effect. If the effect of mechanical interventions is mediated by a change in some biomechanical feature, then it may be that different types (e.g., brace vs. tape), design, or dose of intervention will confer different outcomes via differing influence on the mediating feature (28). Confirming the mediating role that biomechanics may play in mechanical interventions could help to optimize the design and application of mechanical interventions.

Several systematic reviews have evaluated the efficacy of mechanical interventions in knee OA, however very little attention has been given to treatment effect modifiers or mediation analyses (12-21). Moreover, to our knowledge, no study on this topic has yet to pool individual participant data (IPD)

For peer review only - http://bmjopen.bmj.com/site/about/guidelines.xhtml

across studies. An IPD meta-analysis evaluates raw units of data rather than aggregate study-level data, and is thus a more robust approach to evaluating treatment effect modifiers and mediators (29, 30). Compared to traditional study-level meta-analyses, IPD meta-analyses of randomized control trials (RCTs) enhances the ability to handle participant- and study-level confounding, and increases the power to identify responder subgroups and mechanisms underlying treatment effects (30). The results using such an approach may therefore be more reliable and generalizable (30).

Despite the growing recognition of the ethical and scientific importance of data sharing and scientific transparency, one of the biggest challenges in undertaking IPD analyses relates to overcoming data-sharing hurdles (31-33). Barriers range from successfully reaching original study authors; willingness or ability of authors to share data; and international ethics and regulations issues (31-33). The OA Trial Bank is an internationally-recognized organization that was established in 2010 and has developed procedures for navigating these barriers, including safely sharing, handling and storing IPD data from RCTs (34). The OA Trial Bank steering committee supports and approves all projects, including providing input on research questions and study methods. This is therefore the ideal organization in the field of clinical OA research to collaborate with in successfully conducting an IPD meta-analysis.

Aims

We aim to conduct a systematic review and IPD meta-analysis of RCTs, under the guidance of the OA Trial Bank, to evaluate the efficacy of mechanical interventions (i.e., bracing, taping, orthoses, footwear, or canes) in managing knee OA symptoms. Our primary aim is to identify subgroups of individuals with knee OA who respond favourably to mechanical interventions. Our secondary aim is to evaluate the effect of biomechanics as a mediator between mechanical interventions and symptoms.

For peer review only

For peer review only - http://bmjopen.bmj.com/site/about/guidelines.xhtml

Methods and analyses

The OA Trial Bank steering committee approved a summary of this study protocol prior to preparing the full study protocol (34), and we published a basic study protocol with the International Prospective Register of Systematic Reviews (PROSPERO) (35). The current detailed protocol has been written in accordance with the Preferred Reporting Items for Systematic Reviews and Meta-Analysis Protocols (PRISMA-P) (36, 37) statement, and PRISMA Individual Participant Data (PRISMA-IPD) guidelines (29). In collaboration with the OA Trial Bank, we will use methods described previously to guide the transfer and use of IPD (38-40), updated recently to adhere to current European data-sharing regulations (41).

We will search for relevant studies in five databases: MEDLINE, Embase, CINAHL, CENTRAL, and Web of Science, with dates from inception to search date. An initial search was completed 21 August 2019, and we will update this search prior to beginning data analyses. We will develop a search strategy in collaboration with an Erasmus MC librarian, using key words and medical subject headers (MeSH), and adapting the syntax to the respective indexing vocabularies of each database (see Appendix 1).

We will identify studies that meet the following eligibility criteria:

Participants: Adults (18 years or older) with knee OA (tibiofemoral or patellofemoral), diagnosed using any common method (e.g., radiographs, MRI, clinical criteria, diagnosis by a health care professional). We will exclude studies where OA is determined by self-report alone. We will include post-traumatic OA, in particular knees with OA secondary to anterior cruciate ligament injury, regardless of whether or not they were previously reconstructed or repaired. We will, however, exclude knees with non-traumatic OA that have undergone major surgical procedures such as tibial osteotomy or total knee arthroplasty. We will exclude rheumatoid or other inflammatory arthropathies. If a study contains a subgroup of

For peer review only - http://bmjopen.bmj.com/site/about/guidelines.xhtml

participants that meet our inclusion criteria, we will include that study if IPD data are retrieved, or if subgroup analyses are reported in the original publication.

Interventions: Any intervention involving use or wear of mechanical devices (e.g., bracing, taping, orthotics, footwear, cane) that is evaluated after more than one day or application of wear/use. We will include studies that combine these interventions with exercise or education/advice.

Comparison: Any non-surgical treatment (e.g. placebo, usual care, any other intervention that does not involve surgery), waiting list, or no treatment.

Outcomes: Our main outcome will be pain at the end of the study-specific primary duration of treatment. Treatment duration will be categorized as short-term (< 4 weeks), medium-term (4 – 12 weeks), and long-term (> 3 months). Outcome measures will also be extracted at additional time-points during treatment. We will not extract outcomes at any time-points that are measured after discontinuation of treatment. Secondary outcomes will include function, quality of life, global perceived change, OA feature severity, biomechanics, and adverse events.

Study design: We will include peer-reviewed RCTs (or quasi-RCTs). We will exclude any other study design (e.g., non-RCTs, pre-post study designs, observational studies). We will also exclude RCTs that only measure the acute effects of a single application of treatment.

Languages: English, Dutch, German, French

For peer review only

For peer review only - http://bmjopen.bmj.com/site/about/guidelines.xhtml

Titles and abstracts will be initially imported into EndNote X9 (Clarivate Analytics) for deduplication, and then imported into Covidence for screening (42). Two independent reviewers (EMM, MJC) will screen titles and abstracts of all studies identified through this search strategy (43). A third reviewer (MvM) will be consulted in the event of unresolved disagreements. Following the initial screening, two reviewers (EMM, MJC) will independently review full text manuscripts to identify studies for inclusion in this review. A third reviewer (MvM) will again be consulted in the event of disagreements. We will review reference lists of included studies and relevant reviews for additional eligible studies.

For all included studies, we will contact the corresponding author by e-mail. If a current e-mail address cannot be found or the author does not respond (up to three attempts), we will attempt to reach them by other means (e.g. phone, post, contact institution). Where IPD are available and authors or institutions are willing to share data, a data delivery agreement will be signed by both parties. Where local ethics regulations require it, ethics approval will be sought prior to sharing data. Pseudonymized or anonymized data sets (all formats are acceptable, e.g., SPSS, Excel) and related data dictionaries will then be transferred and stored securely on a database at Erasmus MC, for use only as agreed upon in the data delivery agreement. One original study investigator (first or senior author, at the discretion of the data owner) will be invited to be a co-author of the project if they are willing to assume responsibilities that meet authorship guidelines.

We will convert all data sets to a common format, combine data sets with a new variable identifying original trial, and harmonize variables. Data checking will include evaluating baseline characteristics and results of comparisons for our main outcomes against results reported in original publications. We will also check for balancing of baseline participant characteristics in each treatment arm, and evaluate the extent to which all randomized participants in the IPD datasets have been included in study analyses.

For peer review only - http://bmjopen.bmj.com/site/about/guidelines.xhtml

Authors will be consulted in the case of any inconsistencies or discrepancies. In cases where discrepancies cannot be resolved, we will (on a case by case basis) either conduct a sensitivity analysis with that study removed, or we will exclude the study from our analysis altogether.

Data extraction:

Two independent investigators will extract data from all included published studies. From each study, we will extract the following data: country of study; funding source; study design; sample size; target population; inclusion/exclusion criteria; participant characteristics (age, sex, BMI, history of injury or surgery, comorbidities, psychosocial profile, metabolic profile, physical activity/fitness, lifestyle factors, medication use); type, dose and context of intervention (including compliance, co-interventions, protocol deviations, adverse events, drop-out or withdrawal details); OA characteristics (compartment involvement, prevalence, severity, tissues involved), including pre-post if available; and pain, function, quality of life, and biomechanics (e.g. proprioception, knee alignment, strength, kinematics, kinetics) pre-post as available. Global perceived change will also be extracted as available. For all patient-reported outcomes, we will extract recall period in addition to the outcome. For all outcome measures, notably biomechanics, we will also extract whether scores and measures are taken with respect to the device applied/worn or removed. Where IPD are available, we will conduct all analyses using IPD instead of aggregate data, following data consistency checks described above.

Risk of Bias:

Two co-authors will independently evaluate risk of bias (ROB) for each included study using the Cochrane ROB tool version 2 (44), and disagreements will be resolved by a third investigator. The Cochrane ROB v2 considers five domains of possible bias: randomization; deviations from intended interventions; missing outcome data; measurement of the outcome; and selection of the reported

For peer review only - http://bmjopen.bmj.com/site/about/guidelines.xhtml

results. For each domain, ROB is rated as low, some concerns, or high. Both the original publications and IPD datasets will be used for evaluating ROB. The overall study will be considered to be of low ROB if all five domains are rated as low ROB, and high overall ROB if at least one domain is rated as high ROB or if some concerns are identified in multiple domains. We will consult original authors in the event of inadequate reporting or inconsistencies.

Statistical analyses:

We will conduct an IPD meta-analysis of short-term (<4 weeks), medium-term (4 – 12 weeks), and long-term (>3 months) effects of mechanical treatments (alone or in combination with exercise or education/advice) in comparison to other non-surgical treatments, sham, waitlist, or usual care.

Where within-study missing data are sparse (less than 5%), we will assume data are missing completely at random and we will conduct complete case analyses, given the trivial loss of power and negligible implications on bias (45). Where higher proportions of data are missing within a study, we will conduct within-study multiple imputation (45, 46). In cases where a variable was not collected in a given study, we will exclude that study from the relevant analyses.

Treatment efficacy

To evaluate treatment efficacy, we will employ a two-step meta-analysis, first analysing each trial separately, and then pooling results across trials (30, 38, 47). In step one, within each trial, we will evaluate the effect of assigned intervention by intention to treat, regardless of method used in the original study. We anticipate that our main outcome, pain, will be evaluated differently across studies. To navigate this, we will evaluate the pain-related outcome from each study that ranks highest on the recommended hierarchy of pain-related outcomes to be used for meta-analyses (48) (see Table). For

For peer review only - http://bmjopen.bmj.com/site/about/guidelines.xhtml

each available time point (short-, medium-, and long-term), we will fit an ANCOVA model to obtain the treatment effect estimate, including baseline pain as a covariate(49). We will report effect sizes from the ANCOVA model and their respective 95% confidence interval. If study heterogeneity prevents us from harmonizing pain data, then we will navigate this using a statistical approach based on available data. This will likely involve transforming data into standardized means differences (SMDs) or applying proportion of maximum scaling (POMS) methods(50).

In studies where we are unable to obtain IPD, we will extract aggregate data from published manuscripts as they are reported, for example based on final scores or change scores(51). Similar models will be performed for secondary outcomes as data permit. In cases of dichotomous outcomes, we will perform binary modeling and report effect sizes as relative risk (RR, 95% CI).

Table. Hierarchy of pain-related outcomes proposed by Jüni et al 2006(48)

Rank	Pain outcome
Highest	Global pain score (e.g. NRS, VAS)
	Pain on walking (same as 1 but task-specific)
	WOMAC pain subscale(52)
	Other composite pain scores (e.g. KOOS Pain)(53)
	Pain on activities other than walking (e.g. stair climbing)
	WOMAC global score (all 3 subscales combined)
	Lequesne osteoarthritis index global score(54)
	Other algo-functional composite scores
	Patient's global assessment
Lowest	Physician's global assessment

NRS = numeric rating score; VAS = visual analogue scale; WOMAC = Western Ontario McMaster University Osteoarthritis Index; KOOS = Knee injury and Osteoarthritis Outcome Score

In step two, we will perform random effects meta-analysis employing restricted maximum likelihood (REML)(55). We will perform separate meta-analyses for each type of intervention (e.g., braces, taping)

For peer review only - http://bmjopen.bmj.com/site/about/guidelines.xhtml

For peer review only

(30, 47, 56). We will report study heterogeneity as I^2 and τ^2 (57, 58). In cases of notable heterogeneity (I^2 > 50%)(58), we will consider possible sources such as device design, treatment duration, comparison treatment, treatment adherence, or study quality. We will then consider performing meta-regression, subgroup analysis, or sensitivity analyses to explain or account for these potential sources of heterogeneity. We will pool results of studies both with and without IPD data after verifying that effect sizes of IPD studies do not differ from non-IPD studies (30, 51).

In addition to a two-step meta-analysis, we will also perform a one-step meta-analysis as a sensitivity analysis. In a one-step meta-analysis, all IPD datasets are harmonized into one large dataset and analysed together, with the addition of a covariate indicating original trial.

Within studies for which we have IPD and that report adherence to treatment, we will evaluate correlations between adherence and treatment effects. Where IPD are not available, we will extract aggregate data if reported. While we expect clinical and statistical heterogeneity to prevent meaningful meta-analysis of these data, we will pool data where possible.

Treatment effect-modifier analyses

We will conduct treatment effect-modifier analyses to identify subgroups of individuals with knee OA who respond to various mechanical interventions (38). We have proposed several subgroup characteristics that we hypothesize may modify the effect of mechanical interventions on our main outcome, based on expert opinion. These proposed subgroups include the following baseline characteristics: (i) mild vs. severe OA (more severe joint space narrowing is associated with joint malalignment which may respond differently to mechanical interventions); (ii) location of OA, specifically tibiofemoral vs. patellofemoral OA, medial vs. lateral tibiofemoral OA, or medial vs. lateral patellofemoral OA (location of cartilage damage may be associated with differing joint alignment or

For peer review only - http://bmjopen.bmj.com/site/about/guidelines.xhtml

source of symptoms); (iii) varus vs. valgus knee alignment (may be associated with different localized joint forces); (iv) obese vs. non-obese (may confer different amounts of mechanical stress); and (v) post-traumatic vs. non-traumatic OA (possible different biomechanical anomalies). Where feasible, we will apply a two-stage approach, whereby we first investigate within-trial interactions within each study using IPD data, then pool results across trials(27, 56). This approach separates within-trial variation from across-trial variation, thus reducing the risk of ecological bias by analyzing the effect of interest for individuals rather than groups of individuals (27). We will conduct all treatment effect-modifier analyses in IPD data only (56).

Mediation analyses

We hypothesize that biomechanical factors may mediate the effect of these interventions (e.g., kinematics, kinetics, proprioception, hypermobility) by reducing or normalizing joint forces, which in turn reduces pain. If possible, we will conduct mediation analyses to evaluate this hypothesis (38). We acknowledge that it is rare for studies to evaluate biomechanical variables both before and after treatment, so undertaking this analysis will depend on whether there are sufficient data available in included studies. If such an analysis is possible, we will employ a single mediator model to evaluate the proportion of the total effect of the intervention on pain that is mediated by a change in biomechanics (59).

We will conduct funnel plot analyses where there are at least ten studies for a given intervention, to consider the possible effects of small sample size or publication bias (60). We will summarize the overall level of evidence for each category of intervention using the GRADE approach (61).

All analyses will be performed using Stata 15.1 (StataCorp, USA).

For peer review only - http://bmjopen.bmj.com/site/about/guidelines.xhtml

Patient and public involvement

Patients and the public were not directly involved in the design of this study protocol. The OA Trial Bank advisory board includes patient-members who provide overall input to the OA Trial Bank Steering Committee activities. We will solicit patient involvement through the OA Trial Bank advisory board and also through our institutional patient advisors (www.arthrosegezond.nl) for input in the analysis and interpretation of our study results, and to inform and guide dissemination of our study results.

Ethics and dissemination

No new data will be collected, so de novo ethics approval is not required for our study. The OA Trial Bank has established protocols in place to guide the confidential and secure transfer and use of pseudonymized IPD (38-40) that adheres to current European data-sharing regulations (41). We will collaborate with data deliverers to also adhere to relevant institutional, national or international regulations regarding data sharing and ethics. We will store all IPD datasets on a secure driver in accordance with OA Trial Bank procedures. We will disseminate findings via international conferences, open-source publication in peer-reviewed journals, and summaries posted on websites serving the public and clinicians.

Acknowledgements

We thank Dr. Wichor Bramer at Erasmus MC for assisting us with developing our search terms and managing our search.

For peer review only - http://bmjopen.bmj.com/site/about/guidelines.xhtml

Author contributions

The study was conceptualized by EM Macri, M van Middelkoop and SMA Bierma-Zeinstra. V van Middelkoop and SMA Bierma-Zeinstra provided specific guidance regarding OA Trial Bank procedures. Study design and methodology was performed by EM Macri, MJ Callaghan, M van Middelkoop, M Hattle, and SMA Bierma-Zeinstra. Statistical expertise was provided by M Hattle. EM Macri led the draft of the manuscript, and MJ Callaghan, M van Middelkoop, M Hattle, and SMA Bierma-Zeinstra all provided substantial intellectually input to the manuscript. All authors approved the final manuscript.

Funding

Dr. Macri was funded by a Canadian Institutes of Health Research (CIHR) Banting Postdoctoral Fellowship. The OA Trial Bank has received funding from ReumaNederland (grant number CIO-01).

Competing interests

SMABZ reports grants from The Netherlands Organisation for Health Research and Development, CZ, European Union, Foreum, Dutch Arthritis Association, personal fees from Osteoarthritis Research Society International (OARSI), personal fees from Infirst Healthcare, and personal fees from Pfizer, all outside of the submitted work. We have no other disclosures.

References

1. Pereira D, Peleteiro B, Araujo J, Branco J, Santos RA, Ramos E. The effect of osteoarthritis definition on prevalence and incidence estimates: a systematic review. Osteoarthritis Cartilage. 2011;19(11):1270-85.

For peer review only - http://bmjopen.bmj.com/site/about/guidelines.xhtml

2. Guccione AA, Felson DT, Anderson JJ, Anthony JM, Zhang Y, Wilson PW, et al. The effects of specific medical conditions on the functional limitations of elders in the Framingham Study. Am J Public Health. 1994;84(3):351-8.

3. Bannuru RR, Osani MC, Vaysbrot EE, Arden NK, Bennell K, Bierma-Zeinstra SMA, et al. OARSI guidelines for the non-surgical management of knee, hip, and polyarticular osteoarthritis. Osteoarthritis Cartilage. 2019;27(11):1578-89.

4. Beswick AD, Wylde V, Gooberman-Hill R, Blom A, Dieppe P. What proportion of patients report long-term pain after total hip or knee replacement for osteoarthritis? A systematic review of prospective studies in unselected patients. BMJ Open. 2012;2(1):e000435.

5. Hawker GA, Badley EM, Croxford R, Coyte PC, Glazier RH, Guan J, et al. A population-based nested case-control study of the costs of hip and knee replacement surgery. Med Care. 2009:732-41.

6. Robertsson O, Dunbar M, Pehrsson T, Knutson K, Lidgren L. Patient satisfaction after knee arthroplasty: a report on 27,372 knees operated on between 1981 and 1995 in Sweden. Acta Orthopaedica Scandinavica. 2000;71(3):262-7.

7. Ackerman IN, Kemp JL, Crossley KM, Culvenor AG, Hinman RS. Hip and Knee Osteoarthritis Affects Younger People, Too. J Orthop Sports Phys Ther. 2017;47(2):67-79.

8. Felson DT. Osteoarthritis as a disease of mechanics. Osteoarthritis Cartilage. 2013;21(1):10-5.

9. Crossley KM, Vicenzino B, Lentzos J, Schache AG, Pandy MG, Ozturk H, et al. Exercise, education, manual-therapy and taping compared to education for patellofemoral osteoarthritis: A blinded, randomised clinical trial. Osteoarthritis Cartilage. 2015;23(9):1457-64.

10. Skou ST, Roos EM, Laursen MB, Rathleff MS, Arendt-Nielsen L, Rasmussen S, et al. Total knee replacement and non-surgical treatment of knee osteoarthritis: 2-year outcome from two parallel randomized controlled trials. Osteoarthritis Cartilage. 2018;26(9):1170-80.

For peer review only

For peer review only - http://bmjopen.bmj.com/site/about/guidelines.xhtml

11. Quicke JG, Runhaar J, van der Windt D, Healey EL, Foster NE, Holden MA. Moderators of the effects of exercise for hip and knee osteoarthritis: a systematic review of subgroup analyses from randomised controlled trials. Osteoarthritis Cartilage. 2020;28:S161.

12. Gohal C, Shanmugaraj A, Tate P, Horner NS, Bedi A, Adili A, et al. Effectiveness of valgus offloading knee braces in the treatment of medial compartment knee osteoarthritis: a systematic review. Sports health. 2018;10(6):500-14.

13. Penny P, Geere J, Smith TO. A systematic review investigating the efficacy of laterally wedged insoles for medial knee osteoarthritis. Rheumatology international. 2013;33(10):2529-38.

14. Raja K, Dewan N. Efficacy of knee braces and foot orthoses in conservative management of knee osteoarthritis: a systematic review. Am J Phys Med Rehabil. 2011;90(3):247-62.

15. Mine K, Nakayama T, Milanese S, Grimmer K. The effectiveness of braces and orthoses for patients with knee osteoarthritis: A systematic review of Japanese-language randomised controlled trials. Prosthetics and orthotics international. 2017;41(2):115-26.

16. Cherian JJ, Jauregui JJ, Leichliter AK, Elmallah RK, Bhave A, Mont MA. The effects of various physical non-operative modalities on the pain in osteoarthritis of the knee. The bone & joint journal. 2016;98(1_Supple_A):89-94.

17. Brouwer RW, Jakma TS, Verhagen AP, Verhaar JA, Bierma-Zeinstra SM. Braces and orthoses for treating osteoarthritis of the knee. Cochrane Database Syst Rev. 2005(1):CD004020.

18. Cudejko T, van der Esch M, van der Leeden M, Roorda L, Pallari J, Bennell K, et al. Effect of soft braces on pain and physical function in patients with knee osteoarthritis: systematic review with meta-analyses. Osteoarthritis Cartilage. 2017;25:S390-S1.

19. Ouyang J-H, Chang K-H, Hsu W-Y, Cho Y-T, Liou T-H, Lin Y-N. Non-elastic taping, but not elastic taping, provides benefits for patients with knee osteoarthritis: systemic review and meta-analysis. Clinical rehabilitation. 2018;32(1):3-17.

For peer review only - http://bmjopen.bmj.com/site/about/guidelines.xhtml

20. Lu Z, Li X, Chen R, Guo C. Kinesio taping improves pain and function in patients with knee osteoarthritis: A meta-analysis of randomized controlled trials. International Journal of Surgery. 2018.

21. Reilly KA, Barker KL, Shamley D. A systematic review of lateral wedge orthotics—how useful are they in the management of medial compartment osteoarthritis? The Knee. 2006;13(3):177-83.

22. Deveza LA, Loeser RF. Is osteoarthritis one disease or a collection of many? Rheumatology. 2018;57(suppl_4):iv34-iv42.

23. Loeser RF, Goldring SR, Scanzello CR, Goldring MB. Osteoarthritis: a disease of the joint as an organ. Arthritis Rheum. 2012;64(6):1697-707.

24. Bierma-Zeinstra S, Verhagen AP. Osteoarthritis subpopulations and implications for clinical trial design. Arthritis Res Ther. 2011;13:213.

25. Sun X, Ioannidis JPA, Agoritsas T, Alba AC, Guyatt G. How to use a subgroup analysis: users' guide to the medical literature. Jama. 2014;311(4):405-11.

26. Hancock M, Herbert RD, Maher CG. A guide to interpretation of studies investigating subgroups of responders to physical therapy interventions. Physical therapy. 2009;89(7):698-704.

27. Fisher DJ, Carpenter JR, Morris TP, Freeman SC, Tierney JF. Meta-analytical methods to identify who benefits most from treatments: daft, deluded, or deft approach? bmj. 2017;356:j573.

28. Kraemer HC. Messages for clinicians: moderators and mediators of treatment outcome in randomized clinical trials. Am J Psychiatry. 2016;173(7):672-9.

29. Stewart LA, Clarke M, Rovers M, Riley RD, Simmonds M, Stewart G, et al. Preferred reporting items for a systematic review and meta-analysis of individual participant data: the PRISMA-IPD statement. Jama. 2015;313(16):1657-65.

30. Riley RD, Lambert PC, Abo-Zaid G. Meta-analysis of individual participant data: rationale, conduct, and reporting. Bmj. 2010;340:c221.

For peer review only

For peer review only - http://bmjopen.bmj.com/site/about/guidelines.xhtml

31. van Middelkoop M, Lohmander S, Bierma-Zeinstra SMA. Sharing data–taming the beast: barriers to meta-analyses of individual patient data (IPD) and solutions. BMJ Publishing Group Ltd and British Association of Sport and Exercise Medicine; 2020.

32. Lo B. Sharing clinical trial data: maximizing benefits, minimizing risk. Jama. 2015;313(8):793-4.

33. Packer M. Data sharing in medical research. British Medical Journal Publishing Group; 2018.

34. The OA Trial Bank. Individual patient data meta-analysis in osteoarthritis research. [cited 2020 29 May]. Available from: http://www.oatrialbank.com/home.

35. PROSPERO International Prospective Register of Systematic Reviews [cited 2014]. Available from: http://www.crd.york.ac.uk/prospero.

36. Moher D, Shamseer L, Clarke M, Ghersi D, Liberati A, Petticrew M, et al. Preferred reporting items for systematic review and meta-analysis protocols (PRISMA-P) 2015 statement. Systematic reviews. 2015;4(1):1.

37. Shamseer L, Moher D, Clarke M, Ghersi D, Liberati A, Petticrew M, et al. Preferred reporting items for systematic review and meta-analysis protocols (PRISMA-P) 2015: elaboration and explanation. Bmj. 2015;349:g7647.

38. Holden MA, Burke DL, Runhaar J, van Der Windt D, Riley RD, Dziedzic K, et al. Subgrouping and TargetEd Exercise pRogrammes for knee and hip OsteoArthritis (STEER OA): a systematic review update and individual participant data meta-analysis protocol. BMJ open. 2017;7(12):e018971.

39. Deveza LA, Bierma-Zeinstra SMA, Van Spil WE, Oo WM, Saragiotto BT, Neogi T, et al. Efficacy of bisphosphonates in specific knee osteoarthritis subpopulations: protocol for an OA Trial Bank systematic review and individual patient data meta-analysis. BMJ open. 2018;8(12).

40. van Middelkoop M, Arden NK, Atchia I, Birrell F, Chao J, Rezende MU, et al. The OA Trial Bank: meta-analysis of individual patient data from knee and hip osteoarthritis trials show that patients with severe

For peer review only - http://bmjopen.bmj.com/site/about/guidelines.xhtml

pain exhibit greater benefit from intra-articular glucocorticoids. Osteoarthritis Cartilage. 2016;24(7):1143-52.

41. Union EPatCotE. General Data Protection Regulation [29 May 2020]. Available from: https://gdpr-info.eu/.

42. Covidence Systematic Review Software Veritas Health Innovation, Melbourne, Australia. Available from: www.covidence.org.

43. Bramer WM, Milic J, Mast F. Reviewing retrieved references for inclusion in systematic reviews using EndNote. Journal of the Medical Library Association: JMLA. 2017;105(1):84.

44. Higgins JPT, Sterne JAC, Savović J, Page MJ, Hrobjartsson A, Boutron I, et al. A revised tool for assessing risk of bias in randomized trials. Chander J, McKenzie J, Boutron I, Welch V, editors: Cochrane Database of Systematic Reviews; 2016.

45. Quartagno M, Carpenter JR. Multiple imputation for IPD meta-analysis: allowing for heterogeneity and studies with missing covariates. Stat Med. 2016;35(17):2938-54.

46. Koopman L, van der Heijden GJMG, Grobbee DE, Rovers MM. Comparison of methods of handling missing data in individual patient data meta-analyses: an empirical example on antibiotics in children with acute otitis media. Am J Epidemiol. 2008;167(5):540-5.

47. Riley RD, Lambert PC, Staessen JA, Wang J, Gueyffier F, Thijs L, et al. Meta-analysis of continuous outcomes combining individual patient data and aggregate data. Stat Med. 2008;27(11):1870-93.

48. Jüni P, Reichenbach S, Dieppe P. Osteoarthritis: rational approach to treating the individual. Best practice & research Clinical rheumatology. 2006;20(4):721-40.

49. Zhang S, Paul J, Nantha-Aree M, Buckley N, Shahzad U, Cheng J, et al. Empirical comparison of four baseline covariate adjustment methods in analysis of continuous outcomes in randomized controlled trials. Clin Epidemiol. 2014;6:227.

50. Moeller J. A word on standardization in longitudinal studies: don't. Front Psychol. 2015;6:1389-93.

For peer review only - http://bmjopen.bmj.com/site/about/guidelines.xhtml

51. Higgins J, Thomas J, Chandler J, Cumpston M, Li T, Page M, et al., editors. Cochrane Handbook for Systematic Reviews of Interventions version 6.0 (updated July 2019): Cochrane; 2019.

52. Bellamy N. WOMAC Osteoarthritis Index. A user's guide. London: University of Western Ontario; 1995.

53. Roos EM, Roos HP, Lohmander LS, Ekdahl C, Beynnon BD. Knee Injury and Osteoarthritis Outcome Score (KOOS)--development of a self-administered outcome measure. J Orthop Sports Phys Ther. 1998;28(2):88-96.

54. Lequesne MG, Mery C, Samson M, Gerard P. Indexes of severity for osteoarthritis of the hip and knee: validation–value in comparison with other assessment tests. Scand J Rheumatol. 1987;16(sup65):85-9.

55. Langan D, Higgins JPT, Jackson D, Bowden J, Veroniki AA, Kontopantelis E, et al. A comparison of heterogeneity variance estimators in simulated random-effects meta-analyses. Research synthesis methods. 2019;10(1):83-98.

56. Fisher DJ. Two-stage individual participant data meta-analysis and generalized forest plots. The Stata Journal. 2015;15(2):369-96.

57. Higgins JPT, Thompson SG, Deeks JJ, Altman DG. Measuring inconsistency in meta-analyses. BMJ. 2003;327(7414):557-60.

58. Higgins JPT, Thompson SG. Quantifying heterogeneity in a meta-analysis. Stat Med. 2002;21(11):1539-58.

59. Lee H, Herbert RD, Lamb SE, Moseley AM, McAuley JH. Investigating causal mechanisms in randomised controlled trials. Trials. 2019;20(1):1-5.

60. Sterne JAC, Sutton AJ, Ioannidis JPA, Terrin N, Jones DR, Lau J, et al. Recommendations for examining and interpreting funnel plot asymmetry in meta-analyses of randomised controlled trials. Bmj. 2011;343:d4002.

For peer review only

For peer review only - http://bmjopen.bmj.com/site/about/guidelines.xhtml

61. Atkins D, Eccles M, Flottorp S, Guyatt GH, Henry D, Hill S, et al. Systems for grading the quality of evidence and the strength of recommendations I: critical appraisal of existing approaches The GRADE Working Group. BMC Health Serv Res. 2004;4(1):38.

For peer review only

For peer review only - http://bmjopen.bmj.com/site/about/guidelines.xhtml

Appendix 1. Search Terms

Medline (OVID)

(Osteoarthritis, Knee/ OR ((Knee Joint/ OR Knee/ OR Patellofemoral Joint/ OR knee function/) AND Osteoarthritis/) OR (((knee* OR femorotib* OR tibiofemor* OR patellofemoral* OR femoropatell* OR gon) ADJ6 (osteoarth* OR arthrit* OR arthrosis OR oa)) OR (femor* ADJ3 (tibi* OR patell*) ADJ6 (osteoarth* OR arthrit* OR arthrosis OR oa)) OR gonarthr*).ab,ti.) AND (Orthotic Devices/ OR Braces/ OR Foot Orthoses/ OR Shoes/ OR Canes/ OR Crutches/ OR Athletic Tape/ OR (orthos* OR orthotic* OR brace* OR bracing* OR ((orthop* OR foot-worn) ADJ3 (support* OR device* OR applian* OR apparat* OR platform*)) OR shoe* OR crutch* OR cane OR canes OR tape OR taping OR kinesiotap* OR KneeBrac* OR ((mechanical* OR biomechan*) ADJ3 (intervent* OR treat* OR therap*)) OR insole* OR sleeve* OR footwear* OR foot-wear* OR walking-stick* OR walking-aid* OR ((elastic* OR adhesi*) ADJ3 bandage*)).ab,ti.) AND (Exp Controlled clinical trial/ OR "Double-Blind Method"/ OR "Single-Blind Method"/ OR "Random Allocation"/ OR (random* OR factorial* OR crossover* OR cross over* OR placebo* OR ((doubl* OR singl*) ADJ blind*) OR assign* OR allocat* OR volunteer* OR trial OR groups).ab,ti.) NOT (exp Animals/ NOT Humans/)

embase.com

('knee osteoarthritis'/de OR 'patellofemoral arthritis'/de OR (('knee pain'/de OR 'knee'/de OR 'patellofemoral joint'/de OR 'knee function'/de) AND osteoarthritis/de) OR 'Knee Injury and Osteoarthritis Outcome'/de OR 'knee arthritis'/de OR (((knee* OR femorotib* OR tibiofemor* OR patellofemoral* OR femoropatell* OR gon) NEAR/6 (osteoarth* OR arthrit* OR arthrosis OR oa)) OR (femor* NEAR/3 (tibi* OR patell*) NEAR/6 (osteoarth* OR arthrit* OR arthrosis OR oa)) OR gonarthr*):ab,ti) AND ('orthosis'/de OR orthotics/de OR brace/de OR 'knee orthosis'/exp OR 'inshoe orthosis'/de OR 'leg orthosis'/de OR 'foot orthosis'/exp OR 'walking orthosis'/de OR 'shoe'/de OR 'cane'/de OR crutch/exp OR 'walking aid'/exp OR 'orthopedic shoe'/exp OR 'athletic tape'/de OR 'kinesiotape'/de OR 'kinesio tape'/de OR 'kinesio taping'/de OR 'kinesiotaping'/de OR 'bandaging technique'/de OR 'elastic adhesive bandage'/de OR (orthos* OR orthotic* OR brace* OR bracing* OR ((orthop* OR foot-worn) NEAR/3 (support* OR device* OR applian* OR apparat* OR platform*)) OR shoe* OR crutch* OR cane OR canes OR tape OR taping OR kinesiotap* OR KneeBrac* OR ((mechanical* OR biomechan*) NEAR/3 (intervent* OR treat* OR therap*)) OR insole* OR sleeve* OR footwear* OR foot-wear* OR walking-stick* OR walking-aid* OR ((elastic* OR adhesi*) NEAR/3 bandage*)):ab,ti) AND ('Controlled clinical trial'/exp OR 'Crossover procedure'/de OR 'Double-blind procedure'/de OR 'Single-blind procedure'/de OR (random* OR factorial* OR crossover* OR (cross NEXT/1 over*) OR placebo* OR ((doubl* OR singl*) NEXT/1 blind*) OR assign* OR allocat* OR volunteer* OR trial OR groups):ab,ti) NOT ([animals]/lim NOT [humans]/lim)

CINAHL EBSCOhost

(Osteoarthritis, Knee+ OR ((Knee Joint+ OR Knee+ OR Patellofemoral Joint+ OR knee function+) AND Osteoarthritis+) OR (((knee* OR femorotib* OR tibiofemor* OR patellofemoral* OR femoropatell* OR gon) N5 (osteoarth* OR arthrit* OR arthrosis OR oa)) OR (femor* N2 (tibi* OR patell*) N5 (osteoarth* OR arthrit* OR arthrosis OR oa)) OR gonarthr*)) AND (Orthotic Devices+ OR Braces+ OR Foot Orthoses+ OR Shoes+ OR Canes+ OR Crutches+ OR Athletic Tape+ OR (orthos* OR orthotic* OR brace* OR bracing* OR ((orthop* OR foot-worn) N2 (support* OR device* OR applian* OR apparat* OR platform*)) OR shoe* OR crutch* OR cane OR canes OR tape OR taping OR kinesiotap* OR KneeBrac* OR ((mechanical* OR biomechan*) N2 (intervent* OR treat* OR therap*)) OR insole* OR sleeve* OR footwear* OR foot-wear* OR walking-stick* OR walking-aid* OR ((elastic* OR adhesi*) N2 bandage*))) AND (MH Controlled clinical trial+ OR "Double-Blind Method+" OR "Single-Blind Method+" OR "Random Allocation+" OR (random* OR factorial* OR crossover* OR cross over* OR placebo* OR ((doubl* OR singl*) N1 blind*) OR assign* OR allocat* OR volunteer* OR trial OR groups)) NOT (MH Animals+ NOT Humans+)

Cochrane CENTRAL registry of trials

((((knee* OR femorotib* OR tibiofemor* OR patellofemoral* OR femoropatell* OR gon) NEAR/6 (osteoarth* OR arthrit* OR arthrosis OR oa)) OR (femor* NEAR/3 (tibi* OR patell*) NEAR/6 (osteoarth* OR arthrit* OR arthrosis OR oa)) OR gonarthr*):ab,ti) AND ((orthos* OR orthotic* OR brace* OR bracing* OR ((orthop* OR foot next worn) NEAR/3 (support* OR device* OR applian* OR apparat* OR platform*)) OR shoe* OR crutch* OR cane OR canes OR tape OR taping OR kinesiotap* OR KneeBrac* OR ((mechanical* OR biomechan*) NEAR/3 (intervent* OR treat* OR therap*)) OR insole* OR sleeve* OR footwear* OR foot next wear* OR walking next stick* OR walking next aid* OR ((elastic* OR adhesi*) NEAR/3 bandage*)):ab,ti)

Web of science Core Collection

TS=(((((knee* OR femorotib* OR tibiofemor* OR patellofemoral* OR femoropatell* OR gon) NEAR/5 (osteoarth* OR arthrit* OR arthrosis OR oa)) OR (femor* NEAR/2 (tibi* OR patell*) NEAR/5 (osteoarth* OR arthrit* OR arthrosis OR oa)) OR gonarthr*)) AND ((orthos* OR orthotic* OR brace* OR bracing* OR ((orthop* OR foot-worn) NEAR/2 (support* OR device* OR applian* OR apparat* OR platform*)) OR shoe* OR crutch* OR cane OR canes OR tape OR taping OR kinesiotap* OR KneeBrac* OR ((mechanical* OR biomechan*) NEAR/2 (intervent* OR treat* OR therap*)) OR insole* OR sleeve* OR footwear* OR foot-wear* OR walking-stick* OR walking-aid* OR ((elastic* OR adhesi*) NEAR/2 bandage*))) AND (random* OR factorial* OR crossover* OR cross over* OR placebo* OR ((doubl* OR singl*) N1 blind*) OR assign* OR allocat* OR volunteer* OR trial OR groups))

For peer review only

PRISMA-P (Preferred Reporting Items for Systematic review and Meta-Analysis Protocols) 2015 checklist: recommended items to address in a systematic review protocol*

Section and topic	Item No	Checklist item	Page Reported
**ADMINISTRATIVE INFORMATION**			
Title:			
Identification	1a	Identify the report as a protocol of a systematic review	1
Update	1b	If the protocol is for an update of a previous systematic review, identify as such	
Registration	2	If registered, provide the name of the registry (such as PROSPERO) and registration number	2
Authors:			
Contact	3a	Provide name, institutional affiliation, e-mail address of all protocol authors; provide physical mailing address of corresponding author	1
Contributions	3b	Describe contributions of protocol authors and identify the guarantor of the review	15
Amendments	4	If the protocol represents an amendment of a previously completed or published protocol, identify as such and list changes; otherwise, state plan for documenting important protocol amendments	n/a
Support:			
Sources	5a	Indicate sources of financial or other support for the review	15
Sponsor	5b	Provide name for the review funder and/or sponsor	
Role of sponsor or funder	5c	Describe roles of funder(s), sponsor(s), and/or institution(s), if any, in developing the protocol	
**INTRODUCTION**			
Rationale	6	Describe the rationale for the review in the context of what is already known	4-6
Objectives	7	Provide an explicit statement of the question(s) the review will address with reference to participants, interventions, comparators, and outcomes (PICO)	6
**METHODS**			
Eligibility criteria	8	Specify the study characteristics (such as PICO, study design, setting, time frame) and report characteristics (such as years considered, language, publication status) to be used as criteria for eligibility for the review	7-8
Information sources	9	Describe all intended information sources (such as electronic databases, contact with study authors, trial registers or other grey literature sources) with planned dates of coverage	7
Search strategy	10	Present draft of search strategy to be used for at least one electronic database, including planned limits, such that it could be repeated	App 1
Study records:			

For peer review only

Data management	11a	Describe the mechanism(s) that will be used to manage records and data throughout the review	
Selection process	11b	State the process that will be used for selecting studies (such as two independent reviewers) through each phase of the review (that is, screening, eligibility and inclusion in meta-analysis)	8-10
Data collection process	11c	Describe planned method of extracting data from reports (such as piloting forms, done independently, in duplicate), any processes for obtaining and confirming data from investigators	
Data items	12	List and define all variables for which data will be sought (such as PICO items, funding sources), any pre-planned data assumptions and simplifications	
Outcomes and prioritization	13	List and define all outcomes for which data will be sought, including prioritization of main and additional outcomes, with rationale	8,13
Risk of bias in individual studies	14	Describe anticipated methods for assessing risk of bias of individual studies, including whether this will be done at the outcome or study level, or both; state how this information will be used in data synthesis	10
Data synthesis	15a	Describe criteria under which study data will be quantitatively synthesised	9,11-14
	15b	If data are appropriate for quantitative synthesis, describe planned summary measures, methods of handling data and methods of combining data from studies, including any planned exploration of consistency (such as I^2, Kendall's τ)	
	15c	Describe any proposed additional analyses (such as sensitivity or subgroup analyses, meta-regression)	
	15d	If quantitative synthesis is not appropriate, describe the type of summary planned	
Meta-bias(es)	16	Specify any planned assessment of meta-bias(es) (such as publication bias across studies, selective reporting within studies)	14
Confidence in cumulative evidence	17	Describe how the strength of the body of evidence will be assessed (such as GRADE)	14

*** It is strongly recommended that this checklist be read in conjunction with the PRISMA-P Explanation and Elaboration (cite when available) for important clarification on the items. Amendments to a review protocol should be tracked and dated. The copyright for PRISMA-P (including checklist) is held by the PRISMA-P Group and is distributed under a Creative Commons Attribution Licence 4.0.**

From: Shamseer L, Moher D, Clarke M, Ghersi D, Liberati A, Petticrew M, Shekelle P, Stewart L, PRISMA-P Group. Preferred reporting items for systematic review and meta-analysis protocols (PRISMA-P) 2015: elaboration and explanation. BMJ. 2015 Jan 2;349(jan02 1):g7647.

For peer review only
